# Supplementary material for: Pb-rich Cu grain boundary sites for selective CO-to-n-propanol electroconversion
Source: Nat Commun. 2023 Aug 12;14:4882. doi: 10.1038/s41467-023-40689-w (PMC10423280; doi:10.1038/s41467-023-40689-w)
Supplement: Supplementary file 1 — Supplementary Information File [file 41467_2023_40689_MOESM1_ESM.pdf]

Supplementary information for

Pb-rich Cu grain boundary sites for selective CO-to-n-propanol electroconversion

Wenzhe Niu[1,8], Zheng Chen[2,8], Wen Guo[1], Wei Mao[3], Yi Liu[1], Yunna Guo[4], Jingzhao Chen[4], Rui Huang[1], Lin Kang[1], Yiwen Ma[1], Qisheng Yan[1], Jinyu Ye[5], Chunyu Cui[1], Liqiang Zhang[4], Peng Wang[3,6], Xin Xu[2,7†], Bo Zhang[1†]

[1]*State Key Laboratory of Molecular Engineering of Polymers, Department of Macromolecular Science, Fudan University, Shanghai 200438, China.*

[2]*Department of Chemistry, MOE Key Laboratory of Computational Physical Sciences, Shanghai Key Laboratory of Molecular Catalysis and Innovative Materials, Fudan University, Shanghai 200438, China.*

[3]*National Laboratory of Solid State Microstructures, Jiangsu Key Laboratory of Artificial Functional Materials, College of Engineering and Applied Sciences and Collaborative Innovation Center of Advanced Microstructures, Nanjing University, Nanjing 210093, China.*

[4]*Clean Nano Energy Center, State Key Laboratory of Metastable Materials Science and Technology, Yanshan University, Qinhuangdao 066004, China.*

[5]*College of Chemistry and Chemical Engineering, Xiamen University, Xiamen 361005, China.*

[6]*Department of Physics, University of Warwick, Coventry, CV4 7AL, UK.*

[7]*Hefei National Laboratory, Hefei 230088, China.*

[8]*These authors contributed equally: Wenzhe Niu, Zheng Chen.*

†*Correspondence and requests for materials should be addressed to: Bo Zhang (bozhang@fudan.edu.cn) (B.Z.) and Xin Xu (xxchem@fudan.edu.cn) (X.X.).*

Supplementary Notes

Supplementary Note 1

Chemicals. Copper chloride dehydrate (99.99%) was purchased from Macklin. Nano-carbon black (99.5%) was purchased from Aladdin. Potassium hydroxide (≥85.0%), sodium hydroxide (≥96.0%), and methanol (≥99.5%) were purchased from the Sinopharm Chemical Reagent Company. Ethanol (≥99.7%) was purchased from General Reagent. Lead nitrate ($Pb(NO_3)_2$, 99.0%), Cu NPs (25 nm), CuO and Cu_2O were purchased from Sigma-Aldrich. The Nafion solution (Dupont, D-520 dispersion, 5% w/w in water and 1-propanol) was purchased from Alfa Aesar. Freudenberg H23C9 and H14C9 gas diffusion layer (GDL) and Sigracet 39BC GDL were purchased from Fuel Cell Store. A hydroxide exchange membrane FAB-PK-130 was purchased from Fumatech. An anion exchange membrane X37-FA was purchased from Sustainion. All chemicals, including precursors, solvents, hydrophobic agents, and ionomer, unless otherwise stated, were used without further purification.

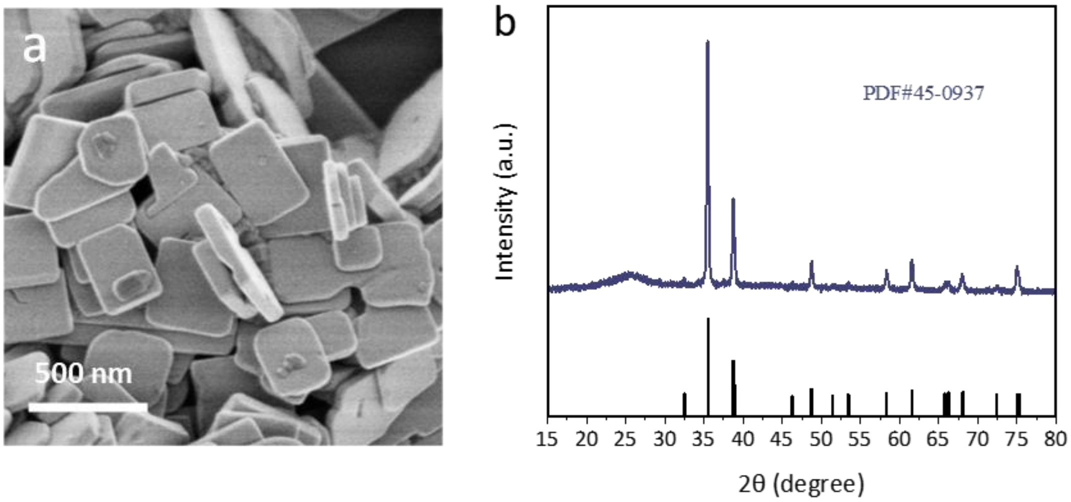

Supplementary Figure 1. Structure analysis of CuO nanopowders. (**a**) SEM and (**b**) XRD patterns of the as-prepared CuO nanopowders, scale bar 500 nm.

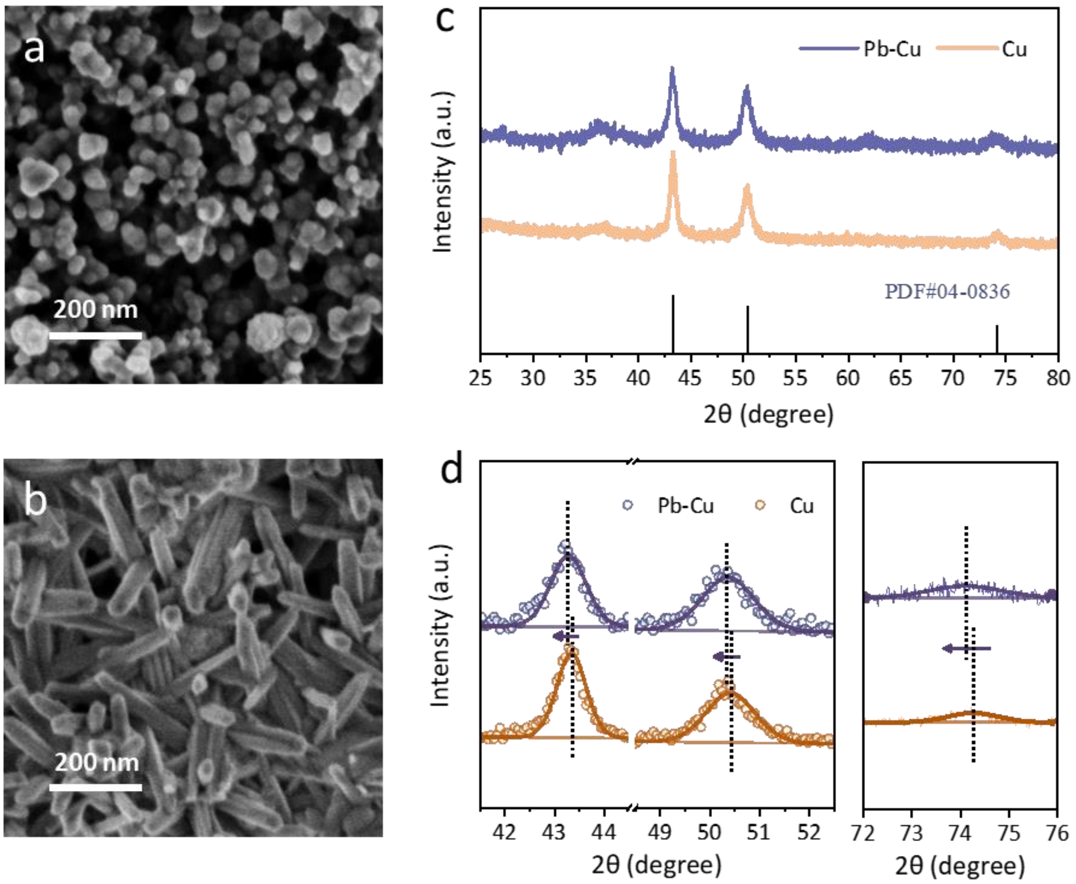

Supplementary Figure 2. Structure analysis of the Pb-Cu and the Cu catalysts. (**a, b**) SEM patterns of the Pb-Cu (**a)** and the Cu **(b**) catalysts, scale bar 200 nm. (**c**) XRD patterns of the Pb-Cu and the Cu catalysts. (**d**) The partial magnification and profile fitting of the corresponding diffraction peaks of the (111), (200) and (220) facets in XRD patterns. The existence of Cu_2O characteristic peaks were attributed to the partial oxidation of the samples in the air after CORR.

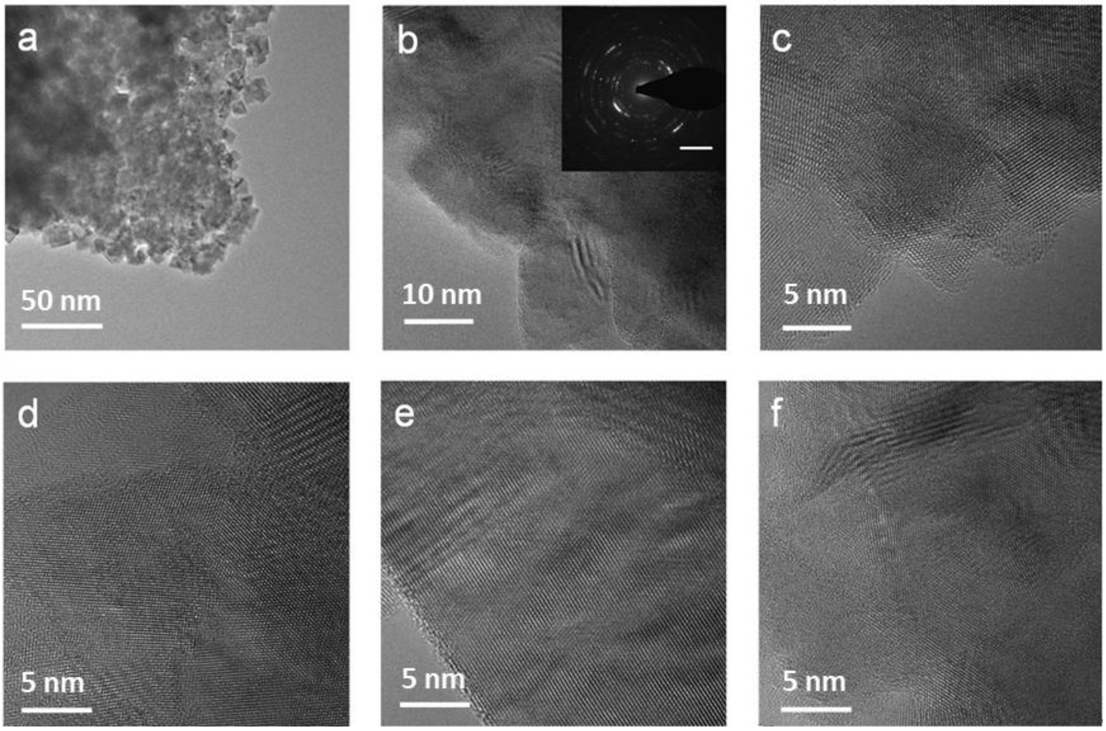

Supplementary Figure 3. TEM images of the Pb-Cu samples. (**a**) TEM images of the Pb-Cu particles. Scale bar, 50 nm. (**b**) HRTEM images of the Pb-Cu particles. Scale bar, 10 nm. Inset, the corresponding SAED pattern. Scale bar, 5 1/nm. (**c-f**) Additional TEM images of the Pb-Cu samples taken from 4 random-selected areas. Scale bar, 5 nm.

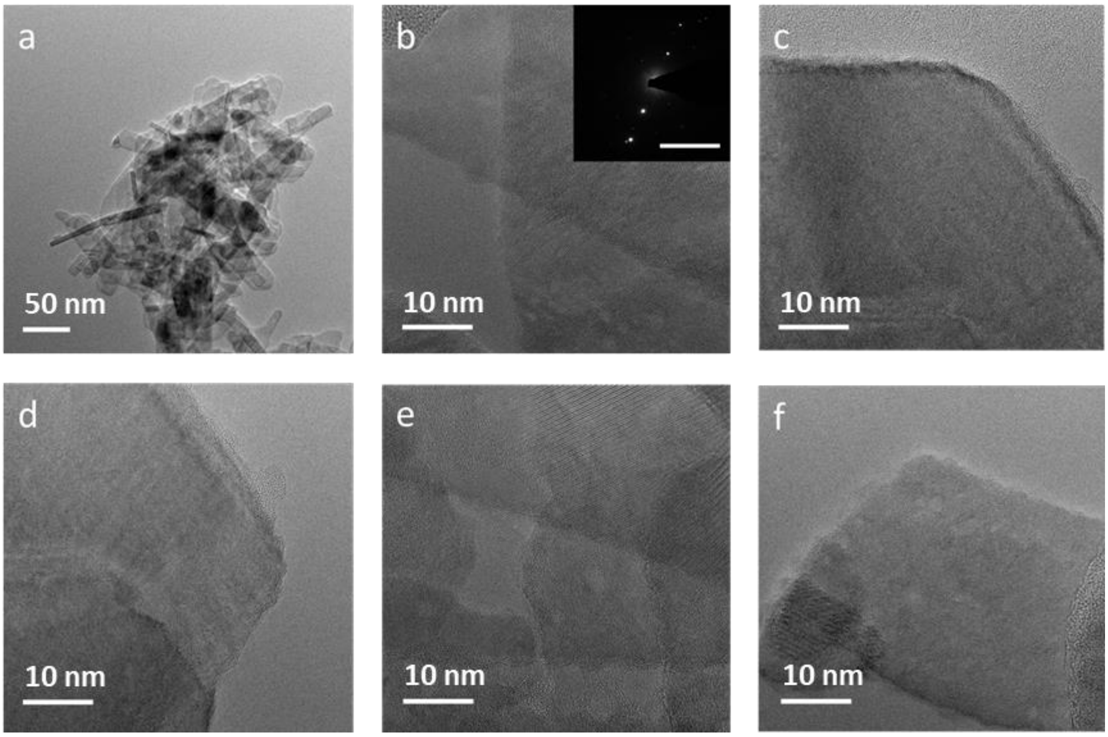

Supplementary Figure 4. TEM images of the Cu samples. (**a**) TEM images of the Cu particles. Scale bar, 50 nm. (**b**) HRTEM images of the Cu particles. Scale bar, 10 nm. Inset, the corresponding SAED pattern. Scale bar, 5 1/nm. (**c-f**) Additional TEM images of the Cu samples taken from 4 random-selected areas. Scale bar, 10 nm.

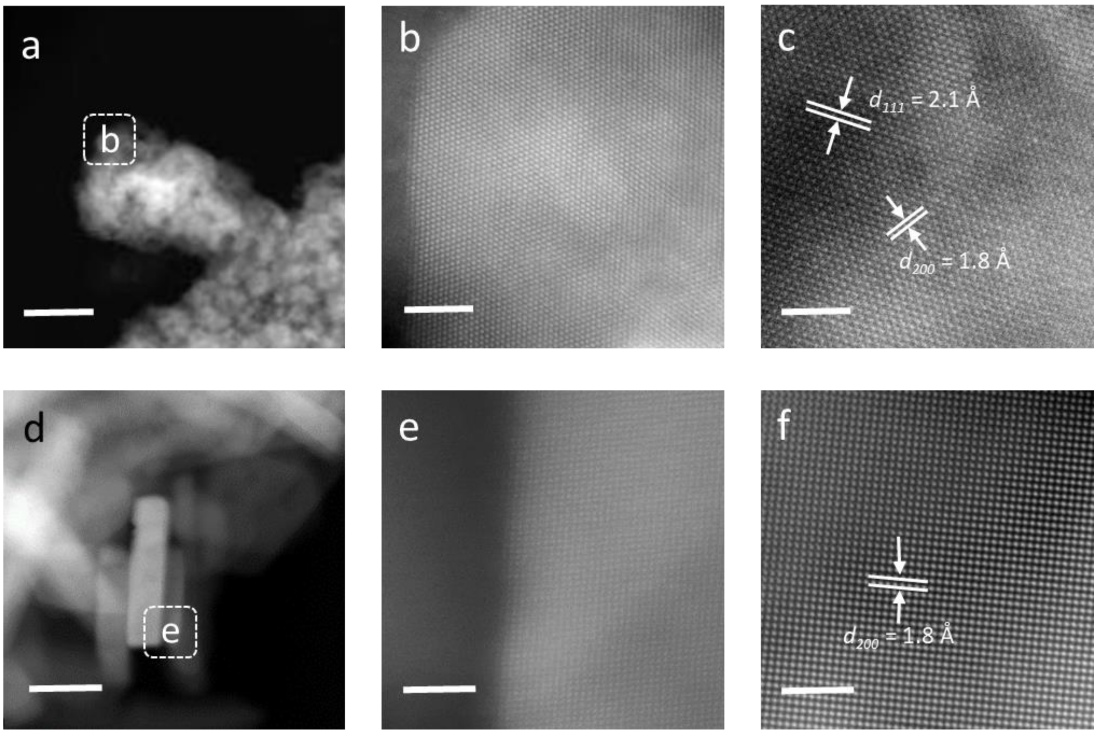

Supplementary Figure 5. HAADF-STEM images of the Pb-Cu and the Cu samples. (**a**, **d**) High-angle annular dark-field scanning transmission electron microscopy (HAADF-STEM) images taken from the edge of nanoparticles of the Pb-Cu (**a**) and the Cu (**d**) samples. Scale bar, 20 nm. (**b**, **c**, **e**, **f**) Atomic-resolution HAADF-STEM images taken from the boxes in (**a**, **d**). Scale bar, 5 nm (**b**, **e**) and 2nm (**c**, **f**).

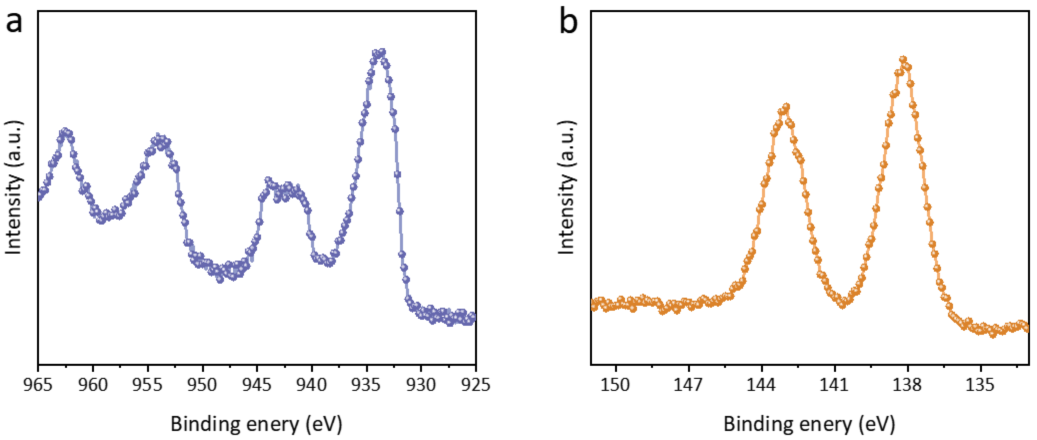

Supplementary Figure 6. High-resolution XPS spectra of the Pb-Cu samples. (**a**) Cu 2*p*. (**b**) Pb 4*f*. The concentration of Pb atoms in the near surface of the Pb-Cu catalyst is approximately 8%. The existences of oxidative states of elements were attributed to partial oxidation of Cu and Pb in air during the catalysts preparation.

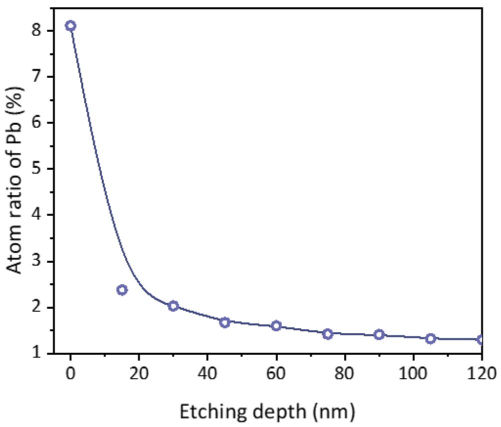

Supplementary Figure 7. In-depth elemental analyses of the Pb-Cu electrode via Ar^+ sputtering XPS. These suggest that Pb atoms mainly exist near the surface of the Pb-Cu catalyst within ~30 nm.

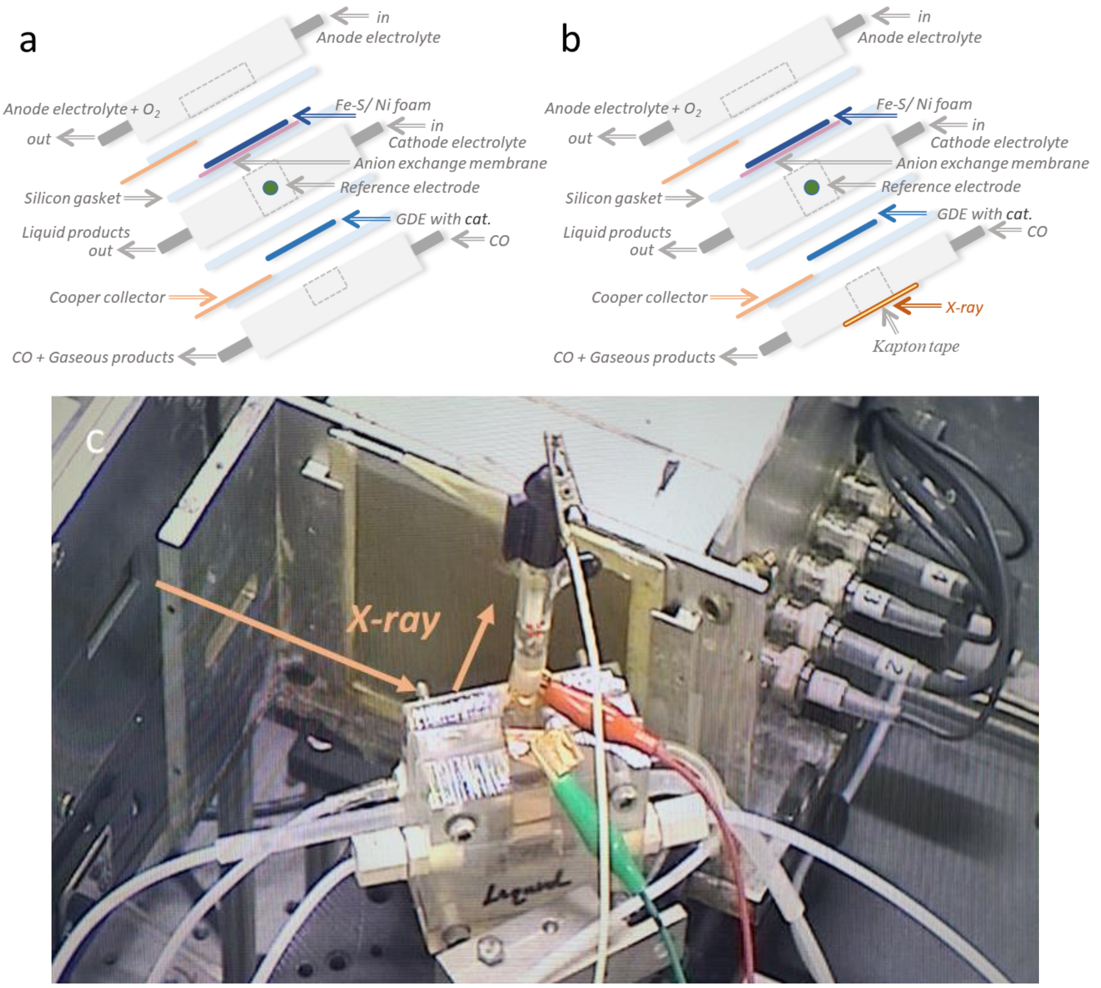

Supplementary Figure 8. Cell configuration of the flow-cell type reactors. A schematic diagram of the flow-cell type electrolyzer configuration for the catalytic performance (**a**) and the operando XAS measurements (**b**). (**c**) An image of the flow-cell type electrolyzer during operando XAFS test.

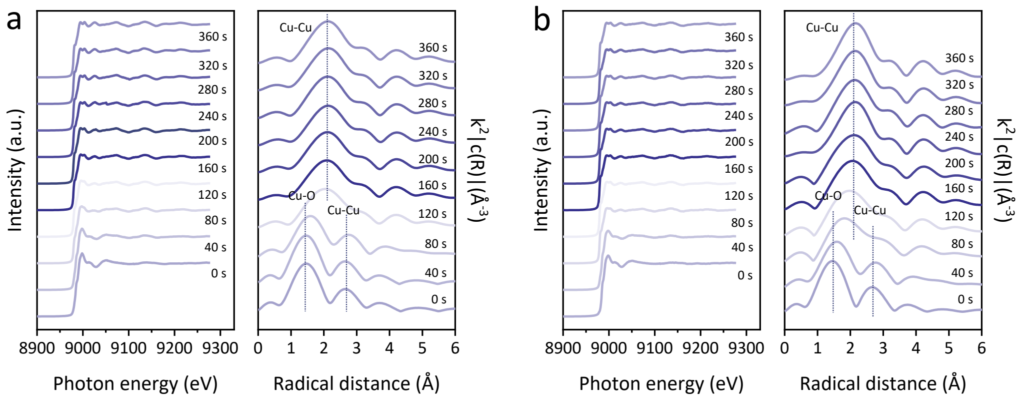

Supplementary Figure 9. Operando Quick-scanning X-ray absorption spectroscopy of different electrodes under the CORR. Operando Cu K-edge XANES (left panels) and EXAFS (right panels) of (**a**) the Pb-Cu and (**b**) the Cu electrodes. We took a note that there is a relatively sluggish Cu^{2+} to Cu^{0} transformation with the Pb^{2+} ions assistance, showing a suppressed Cu reduction with the Pb doping.

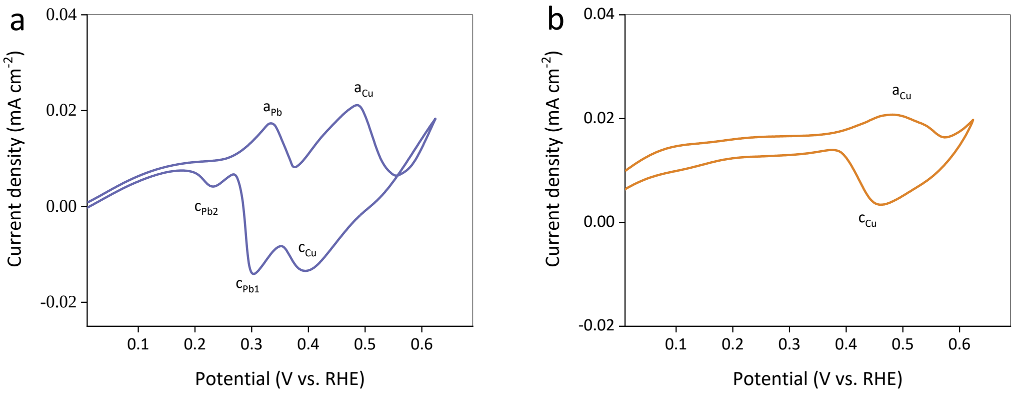

Supplementary Figure 10. Cyclic-voltammetry analyses of the Pb-Cu (a) and the Cu (b) catalysts. The cathodic peak of CuO/Cu shifts to lower potential on Pb-Cu compared to the Cu electrode suggests that Cu reduction was suppressed after Pb-doping. This phenomenon is consistent with Quick-scanning X-ray absorption spectroscopy. The suppressed Cu reduction might be attributed to the electron deficiency of surface Cu atoms in Pb-Cu caused by the more stable Cu-O and Cu-OH bonds caused by the higher electronegativity of Pb relative to Cu[1,2].

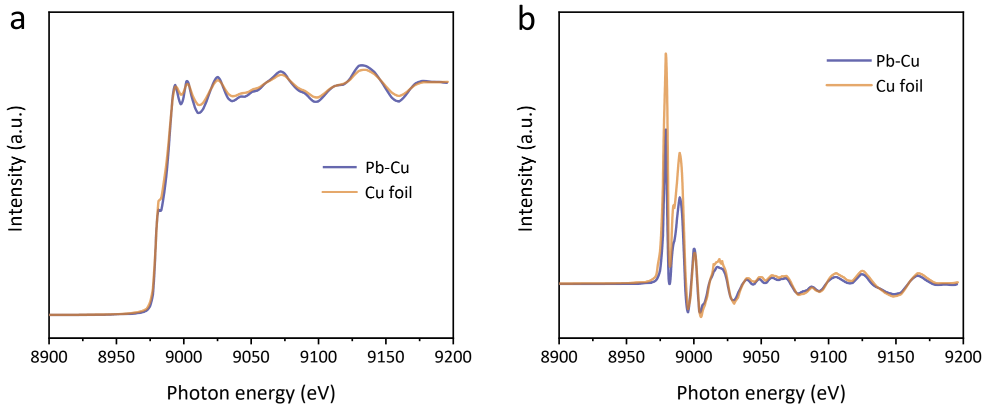

Supplementary Figure 11. Operando Cu K-edge XAS analyses of the Pb-Cu catalysts. (**a**) normalized and (**b**) The first derivatives of the operando Cu K-edge XANES spectra of the Pb-Cu catalysts at -0.68 V (vs. RHE) during CORR. Cu foil was listed for comparison.

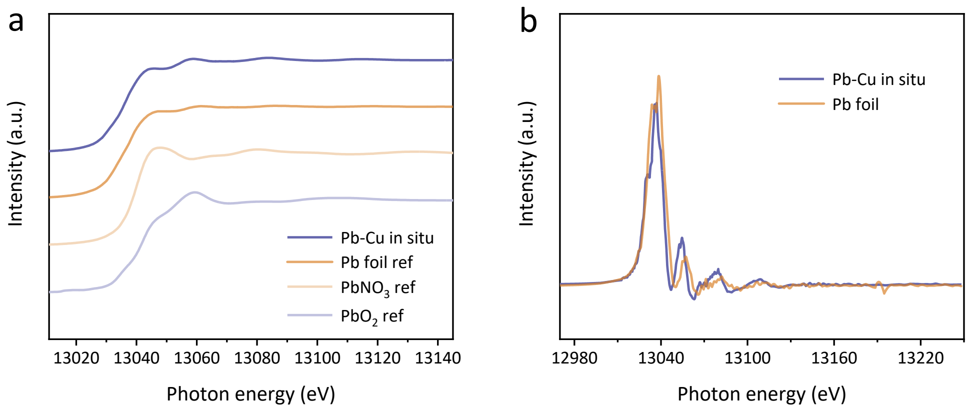

Supplementary Figure 12. Operando Pb L_3-edge XAS analyses of the Pb-Cu catalysts. (**a**) normalized and (**b**) The first derivatives of the operando Pb L_3-edge XANES spectra of the Pb-Cu catalysts at -0.68 V (vs. RHE) during CORR. Pb foil, $Pb(NO_3)_2$ and PbO_2 were listed for comparison.

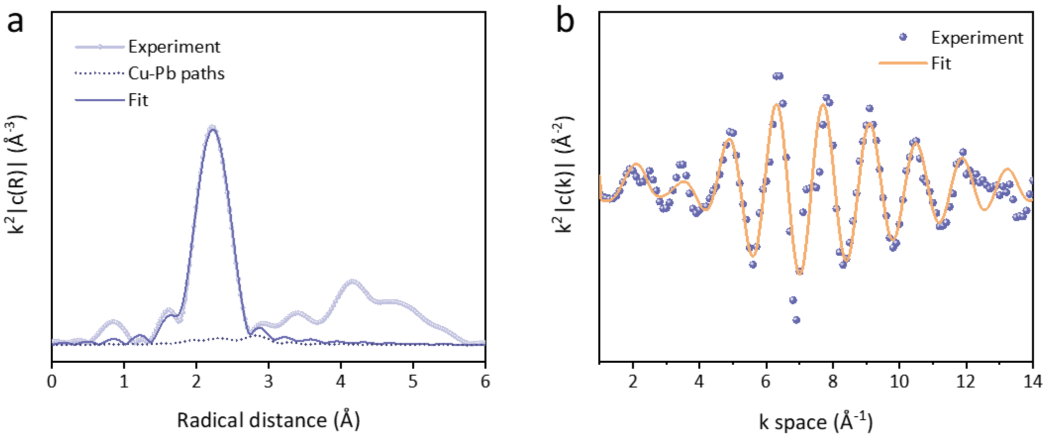

Supplementary Figure 13. Operando Cu K-edge EXAFS analyses of the Pb-Cu catalysts. (**a**) Fourier-transformed $k^2\chi(k)$ of the Pb-Cu catalysts at -0.68 V (vs. RHE) during CORR. Insert, the partial magnification of the Pb-Cu paths. (**b**) The corresponding K space pattern of the Pb-Cu catalysts.

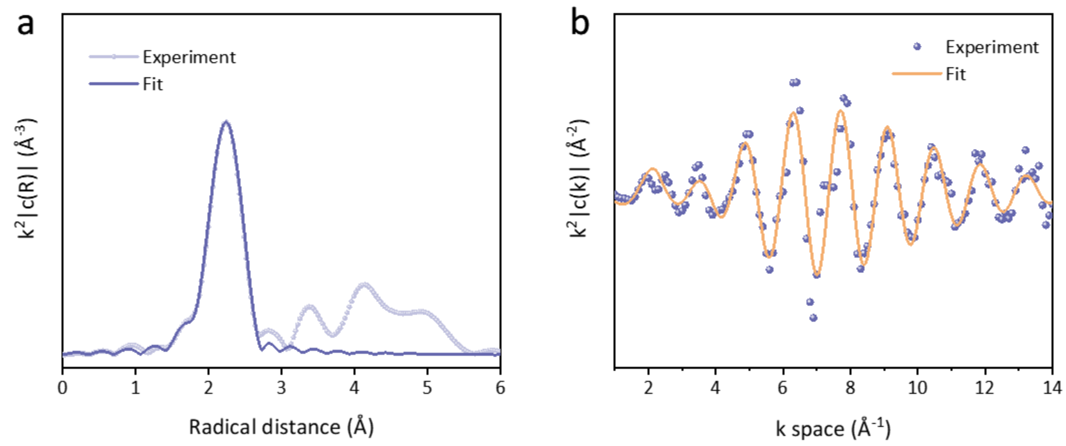

Supplementary Figure 14. Operando Cu K-edge EXAFS analyses of the Cu catalysts. (**a**) Fourier-transformed $k^2\chi(k)$ of the Cu catalysts at -0.68 V (vs. RHE) during CORR. (**b**) The corresponding K space pattern of the Cu catalysts.

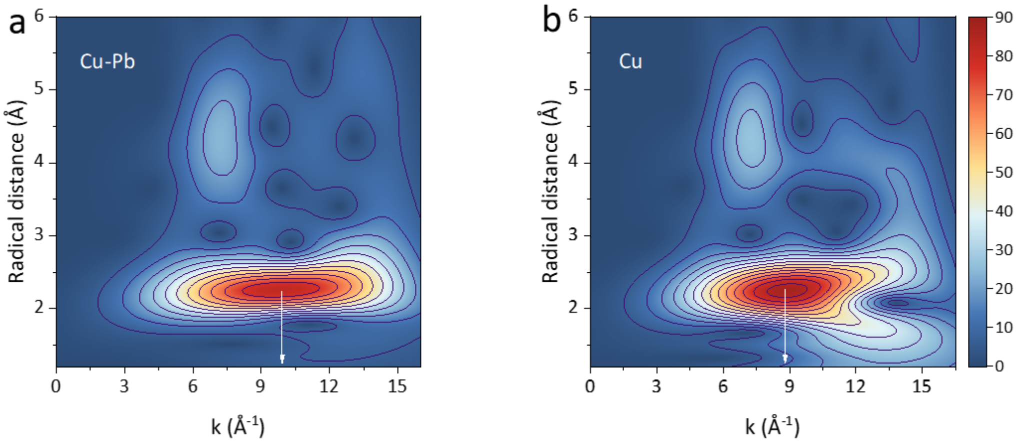

Supplementary Figure 15. WT-EXAFS Cu K-edge spectra for different catalysts. The spectra were obtained on (**a**) the Pb-Cu samples and (**b**) the Cu samples, respectively. The higher k-value of WT maximum for the Pb-Cu catalysts indicates the existence of the Cu-Pb paths.

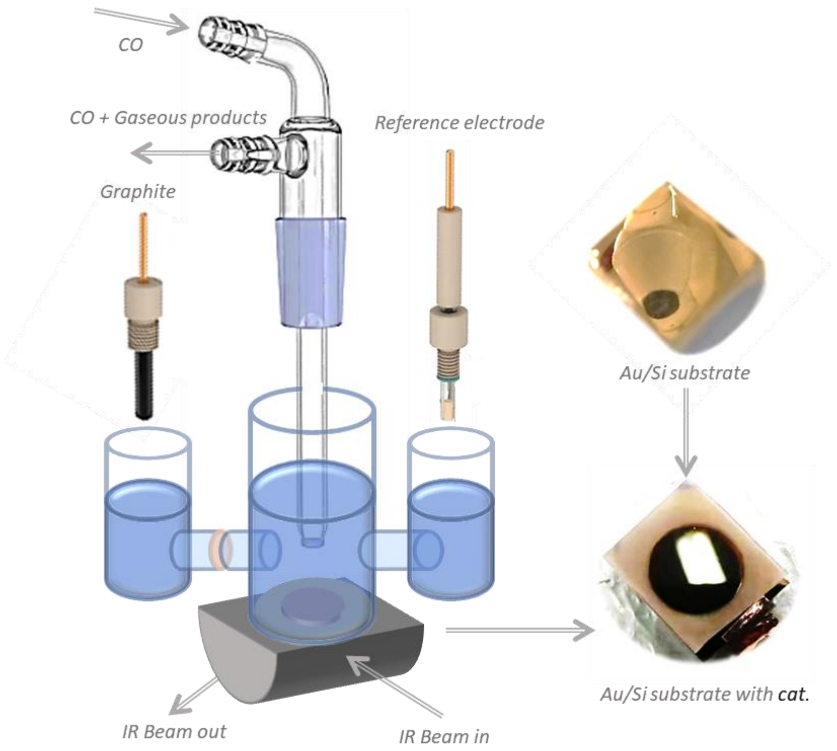

130

131 **Supplementary Figure 16. Cell configuration of the H-type cell for operando ATR-SEIRAS.**

132

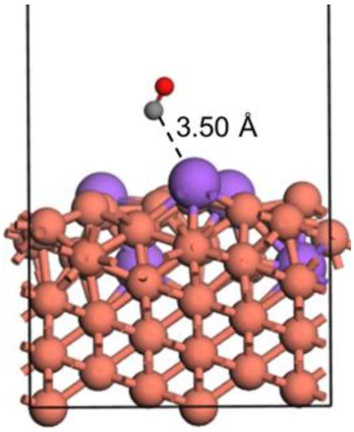

Supplementary Figure 17. The optimized structure for CO adsorption on Pb element of Pb-Cu surface by DFT calculations.

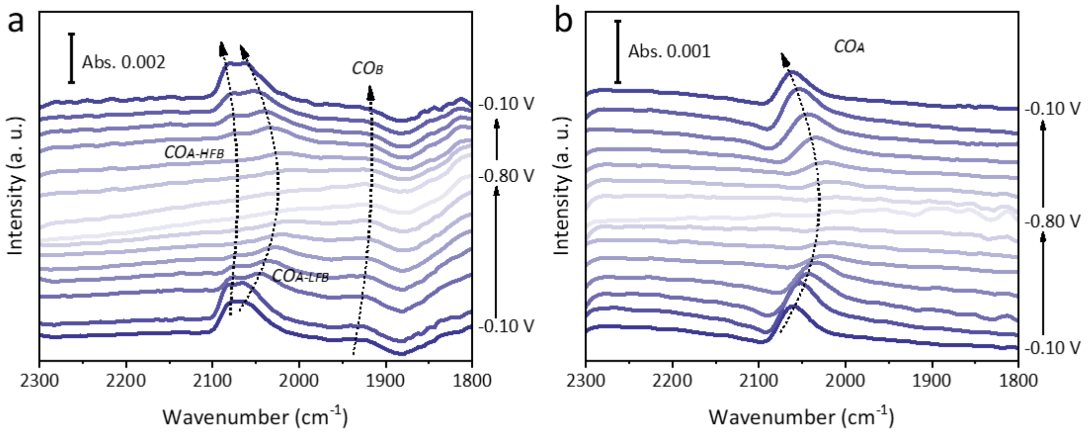

Supplementary Figure 18. The operando ATR-SEIRAS spectra on different electrodes under CORR. The spectra were recorded on the Pb-Cu (**a**) and the Cu (**b**) catalysts with negative staircase potential scan from -0.10 V to -0.80 V and backward to -0.10V (vs. RHE).

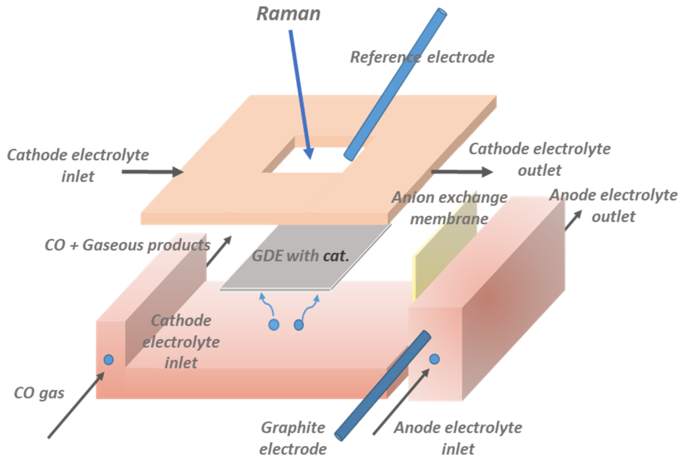

Supplementary Figure 19. Cell configuration of the modified flow-cell type reactors for operando Raman spectroscopy.

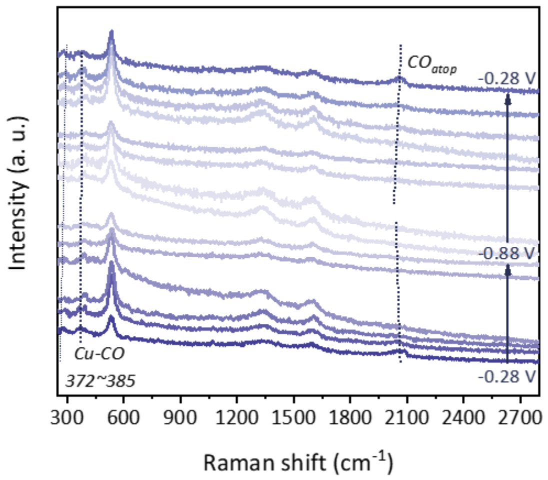

Supplementary Figure 20. The operando Raman spectra on the Cu electrode under CORR. The spectra were recorded on the same point at different potentials from -0.28 V to -0.88 V and backward to -0.28V (vs. RHE).

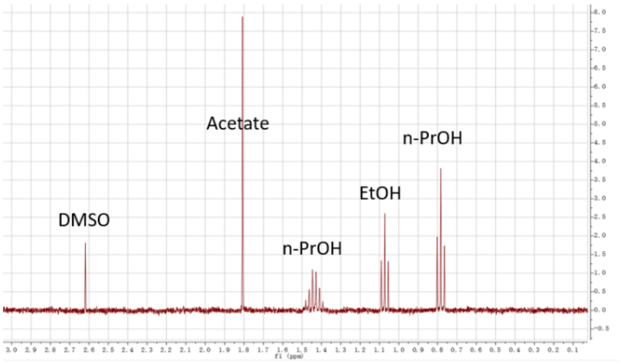

Supplementary Figure 21. NMR spectra of liquid products. ^{1}H NMR spectra of liquid products from the Pb-Cu catalyst under -0.68 V (vs. RHE) after 10 h operation of CORR in the flow cell.

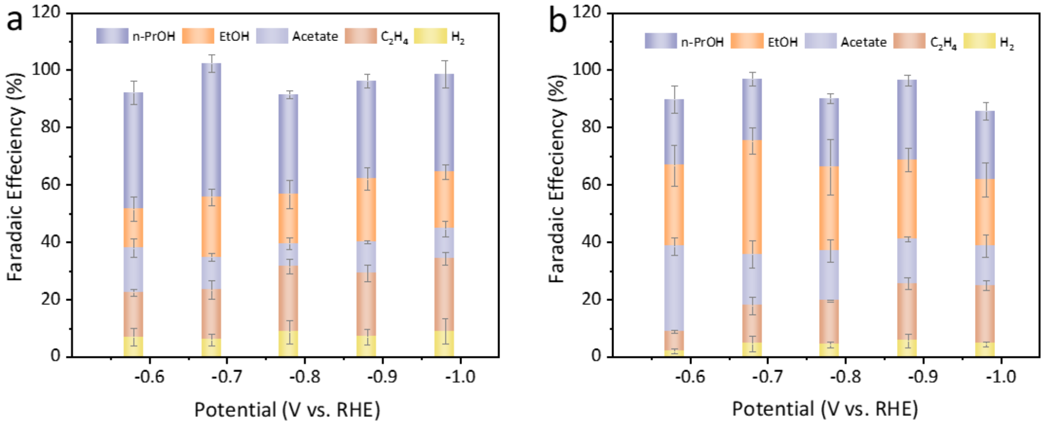

Supplementary Figure 22. CORR performances of catalysts. The FEs of the CORR products on (**a**) the Pb-Cu and (**b**) the Cu catalysts under different potentials in 1M KOH. Error bars based on the standard deviation of at least three independent samples.

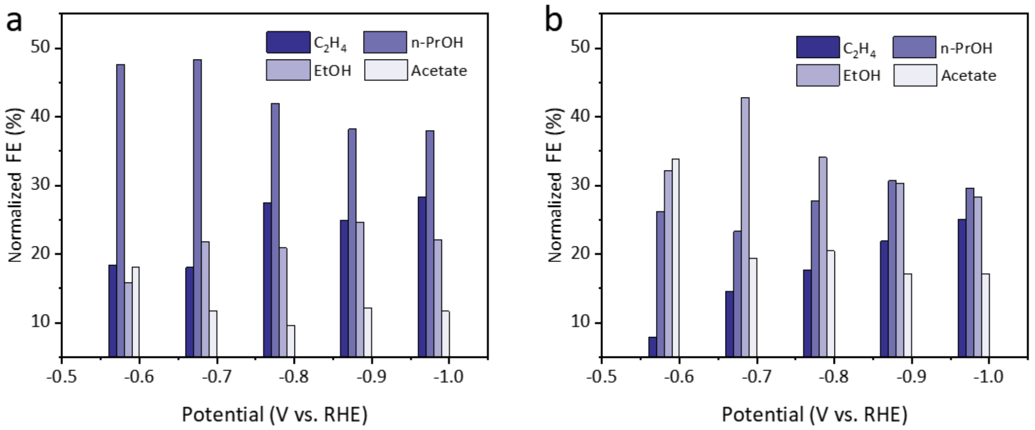

Supplementary Figure 23. Normalized CORR performances[3] of the catalysts. The normalized FEs of the C_{2+} products on (a) the Pb-Cu and (b) the Cu catalysts at different applied potentials in 1 M KOH.

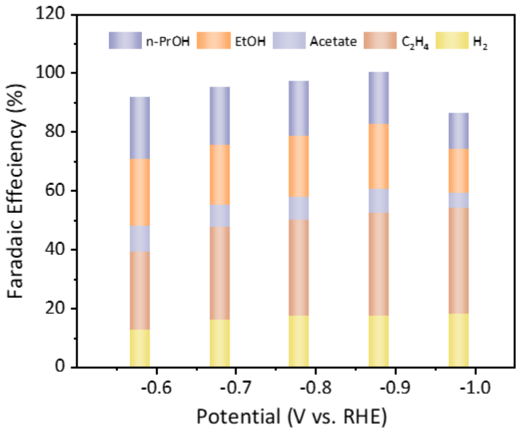

Supplementary Figure 24. CORR performances of the catalyst. The FEs of the CORR products on commercial 20 nm Cu nanoparticles under different potentials in 1M KOH.

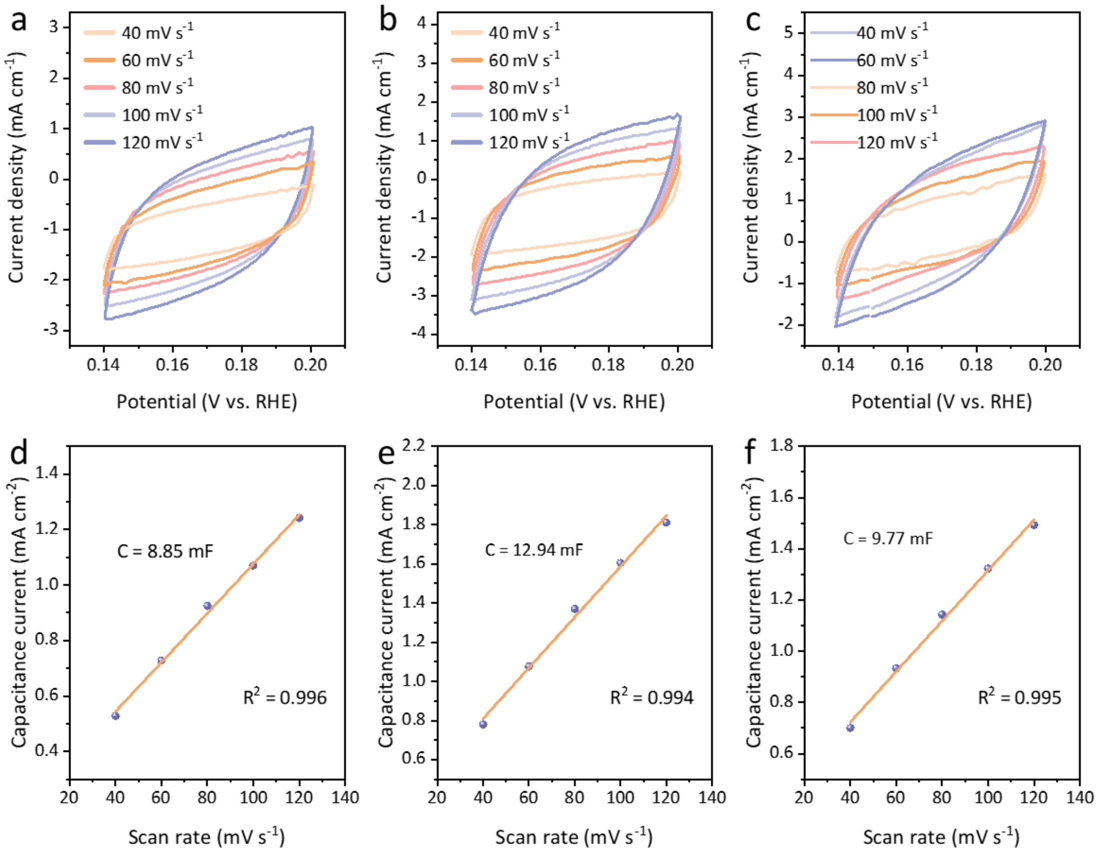

Supplementary Figure 25. Electrochemical surface area measurement (ECSA) for different catalysts. Determination of double-layer capacitances over a range of scan rates for different catalysts in 1 M KOH saturated with Ar: Pb-Cu (**a**, **d**), Cu (**b**, **e**) and Com-Cu (**c**, **f**). The average capacitance current *j* ($j = (j_a - j_c)/2$, where j_a and j_c are anodic and cathodic current densities, respectively) for more than 20 circles at 0.17 V (*vs.* RHE) against the scan rates, C_{dl} values are given by the slopes.

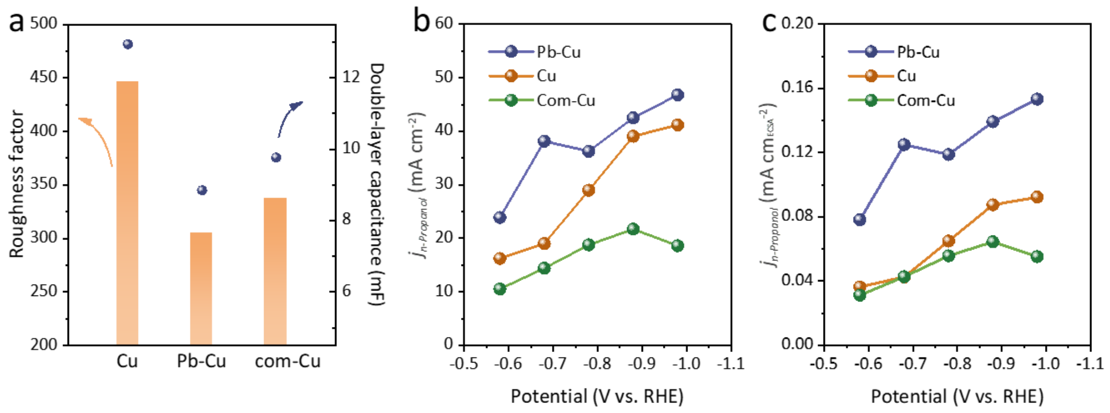

Supplementary Figure 26. CORR performance of different cathode electrodes. (**a**) Electric double-layer capacitances and surface roughness factors of the catalysts, calculated by defining the surface roughness factor for electropolished polycrystalline Cu with an electric double layer capacitance of 29 mF as 1 on different electrodes. (**b**) Partial n-propanol current densities with respect to geometrical areas on different electrodes at various potentials. (**c**) Electrochemically active surface area (ECSA)-normalized partial n-propanol current densities on different electrodes.

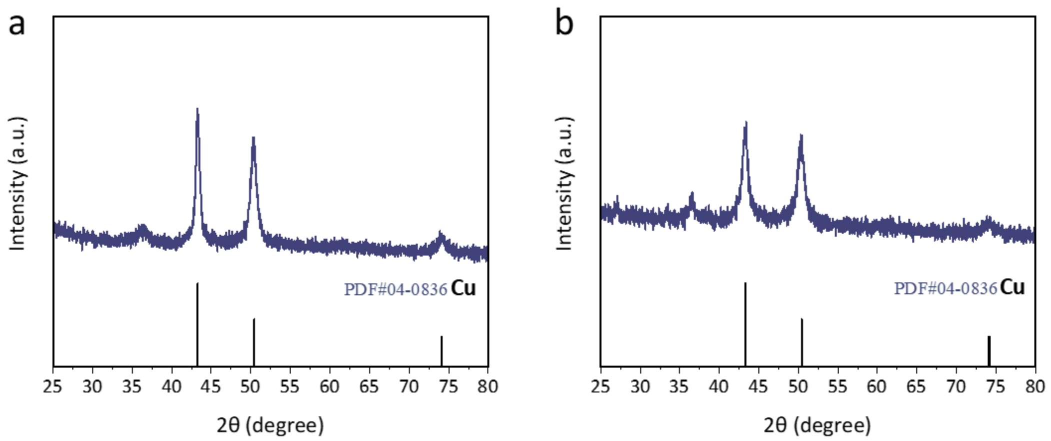

Supplementary Figure 27. XRD patterns of samples with different Pb concentration. The XRD patterns of the Pb-Cu-l (**a**) and the Pb-Cu-h (**b**) catalysts. The existence of Cu_2O characteristic peaks were attributed to the partial oxidation in the air of the samples. No crystalline Pb features were observed on both samples.

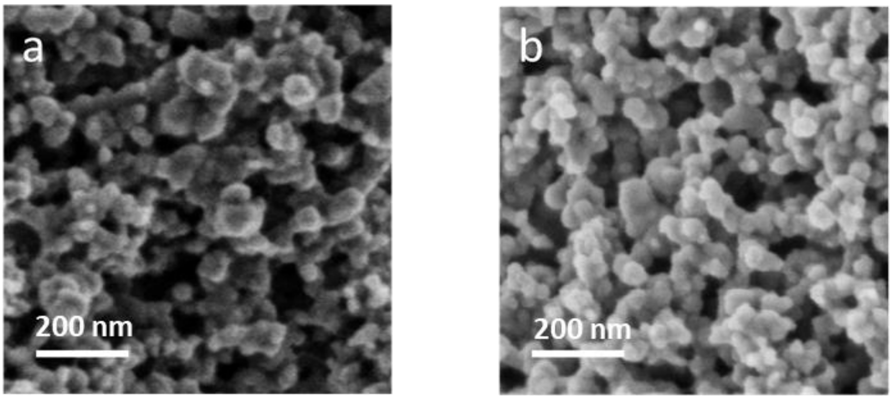

Supplementary Figure 28. SEM images of the Pb-Cu-l and the Pb-Cu-h samples. SEM images of the Pb-Cu-l (**a**) and the Pb-Cu-h (**b**) particles. Scale bar, 200 nm.

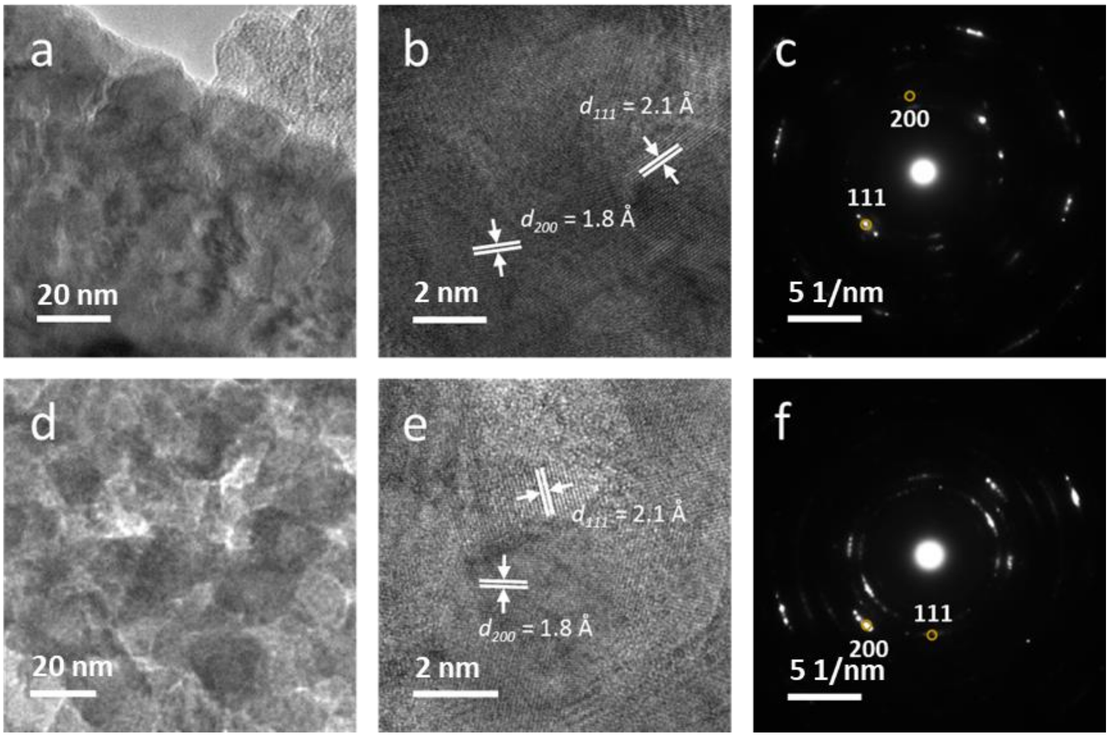

Supplementary Figure 29. TEM images of the Pb-Cu-l and the Pb-Cu-h samples. (**a**, **d**) TEM images of the Pb-Cu-l (**a**) and the Pb-Cu-h (**d**) particles. Scale bar, 20 nm. (**b**, **e**) HRTEM images of the Pb-Cu-l (**b**) and the Pb-Cu-h (**e**) particles. Scale bar, 2 nm. (**c**, **f**) The corresponding SAED patterns of **a** and **d**. Scale bar, 5 1/nm.

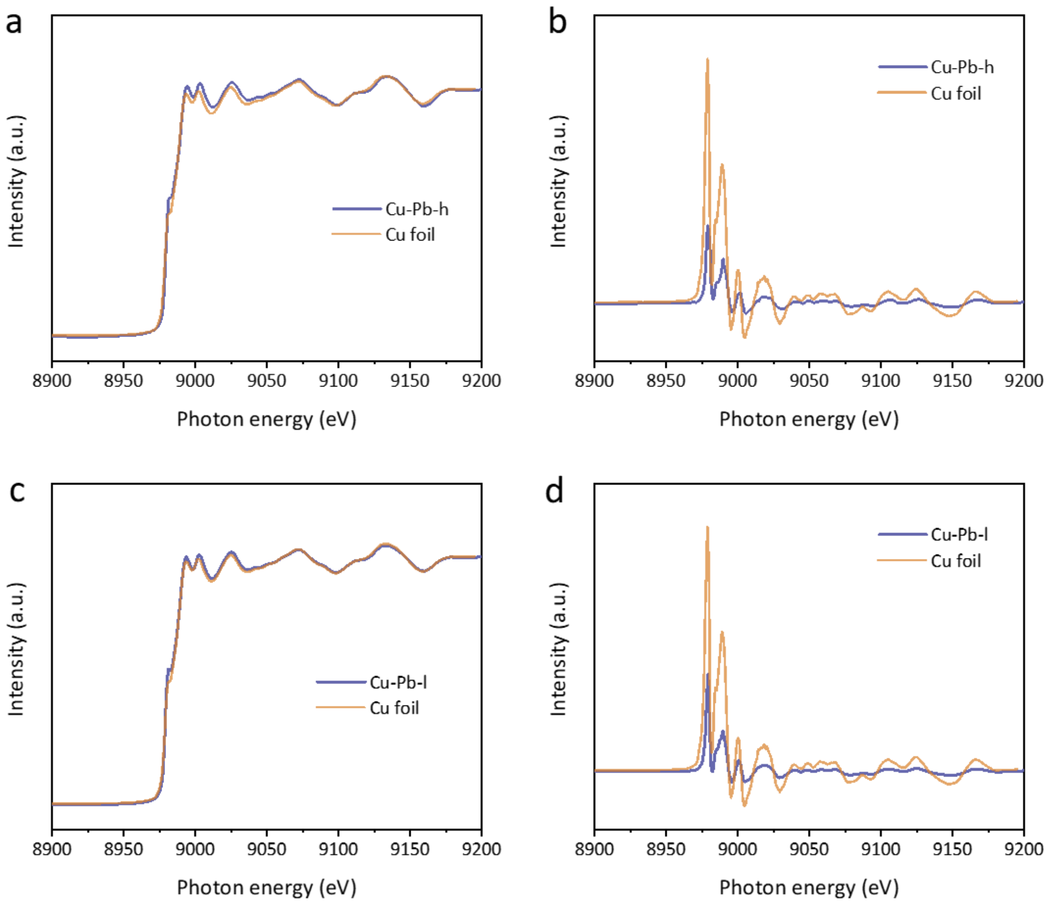

Supplementary Figure 30. Operando Cu K-edge XAS analyses of different catalysts. (**a**, **c**) normalized and (**b**, **d**) The first derivatives of the operando Cu K-edge XANES spectra of the Pb-Cu-l and the Pb-Cu-h catalysts at -0.68 V (vs. RHE) during CORR, respectively. Cu foil was listed for comparison.

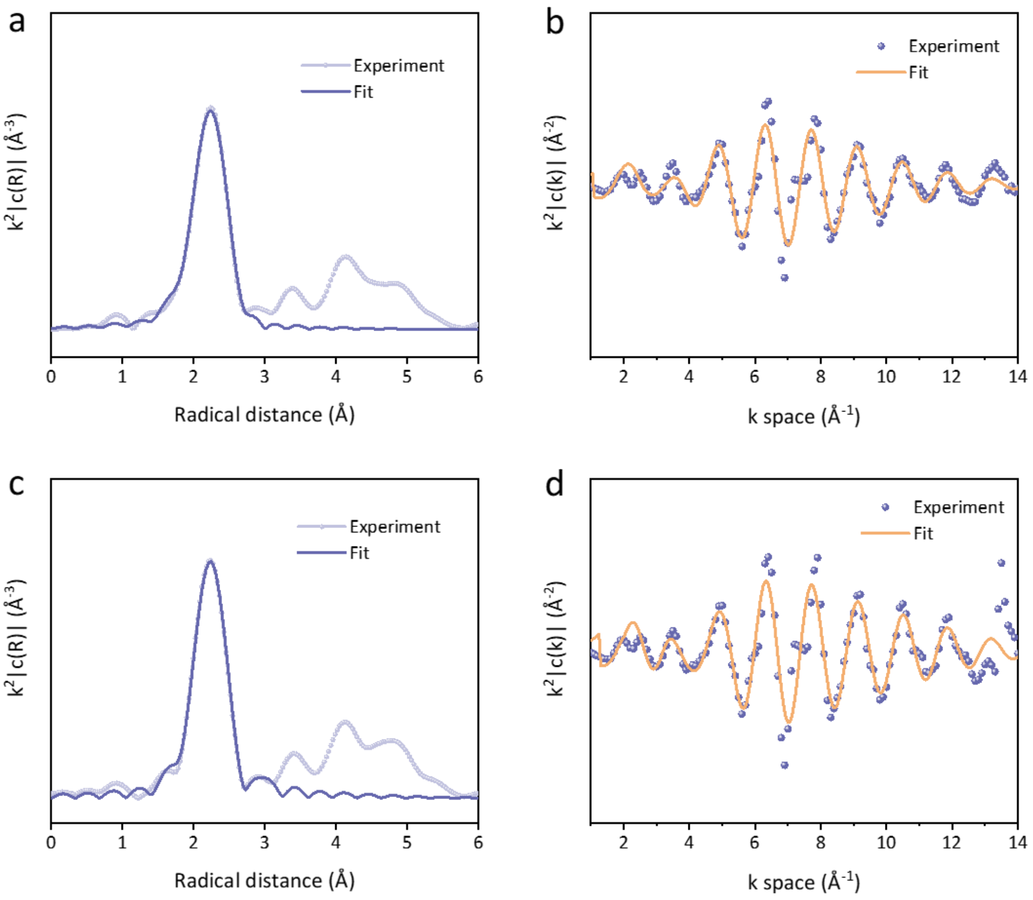

Supplementary Figure 31. Operando Cu K-edge EXAFS analyses of different catalysts. (**a**, **c**) Fourier-transformed $k^2\chi(k)$ of the Pb-Cu-l and the Pb-Cu-h catalysts at -0.68 V (vs. RHE) during CORR. (**b**, **d**) The corresponding K space pattern of the Pb-Cu-l and the Pb-Cu-h catalysts.

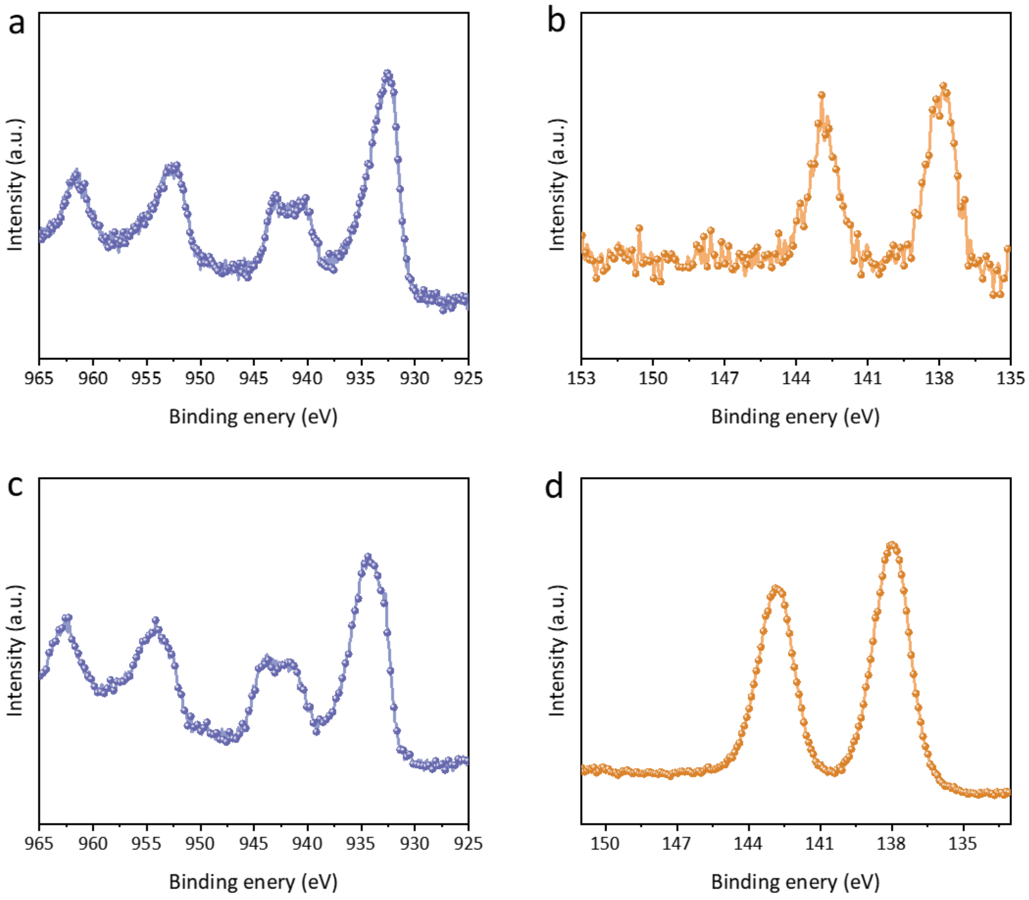

Supplementary Figure 32. High-resolution XPS spectra of the Pb-Cu-l and the Pb-Cu-h catalysts. (**a**, **c**) Cu 2*p* for the Pb-Cu-l (**a**) and the Pb-Cu-h (**c**) catalysts. (**b**, **d**) Pb 4*f* for the Pb-Cu-l (**b**) and the Pb-Cu-h (**d**) catalysts. The concentration of Pb atoms in the near surface of the Pb-Cu-l and the Pb-Cu-h catalysts are approximately 2.3% and 17.0%, respectively. The existences of oxidative states of elements were attributed to partial oxidation of Cu and Pb in air during the catalyst preparation.

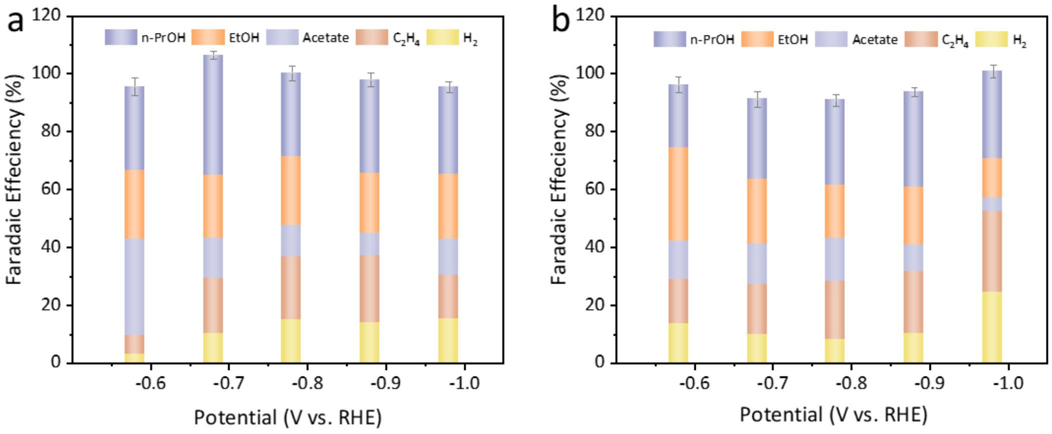

Supplementary Figure 33. CORR performances of catalysts with different Pb concentration. The FEs of CORR products on (**a**) the Pb-Cu-l and (**b**) the Pb-Cu-h catalysts under different potentials in 1M KOH. Error bars based on the standard deviation of at least three independent samples.

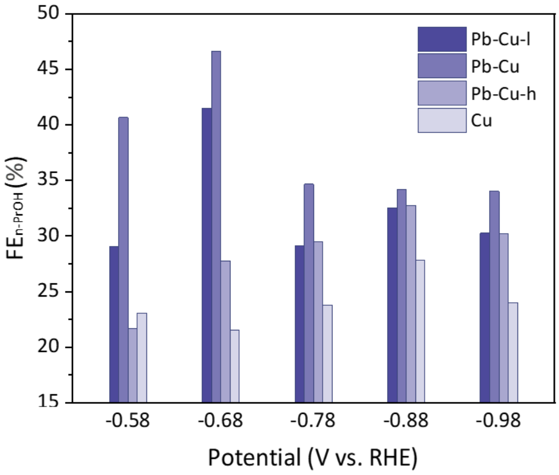

Supplementary Figure 34. Comparison of CO-to-n-propanol FEs for various catalysts.

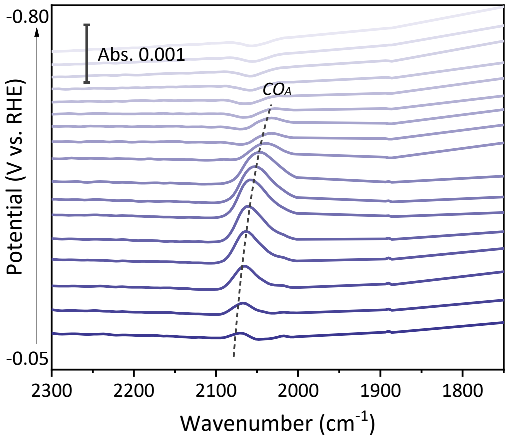

Supplementary Figure 35. Operando electrochemical ATR-SEIRAS spectra. ATR-SEIRAS spectra of 20 nm Cu nanoparticles catalyst under different applied potentials versus RHE using 0.1 M KOH electrolyte during CORR.

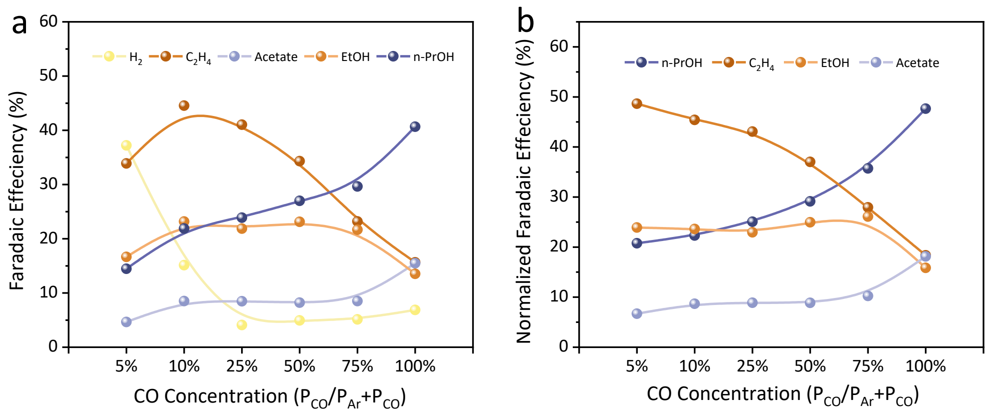

Supplementary Figure 36. CORR performances of the Pb-Cu catalyst under different CO concentration. The FEs (**a**) and normalized FEs[3] (**b**) of the CORR products on Pb-Cu under different CO concentration at -0.68 V (*vs.* RHE) in 1M KOH.

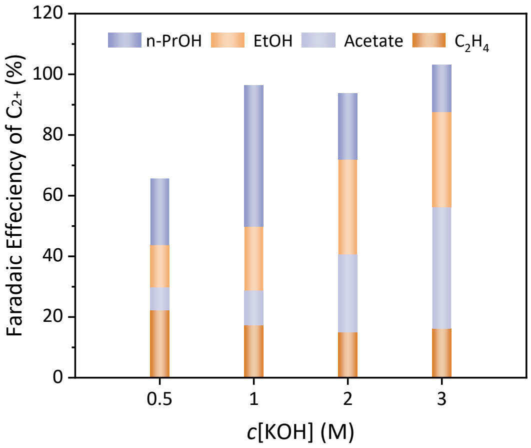

Supplementary Figure 37. CORR performance on the Pb-Cu catalysts in electrolytes with different KOH concentration at -0.68 V (*vs.* RHE with no *i*R compensation).

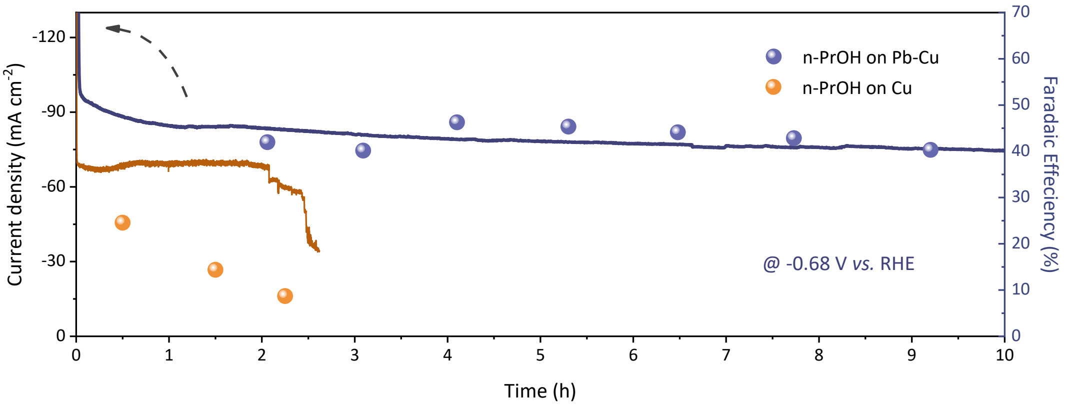

Supplementary Figure 38. Flow-cell stability test of the Pb-Cu and the Cu catalysts for CORR. FEs of n-propanol during 10 hours of CORR test under the constant potential of -0.68 V (*vs.* RHE).

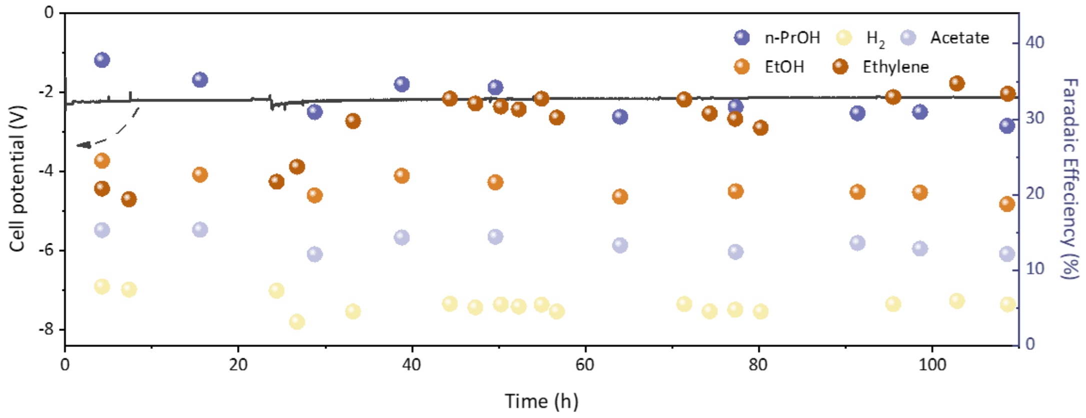

Supplementary Figure 39. Membrane electrode assemble (MEA) stability test of the Pb-Cu catalyst for CORR. Product distribution during 105 hours of CORR test under the constant current of 1.0 A.

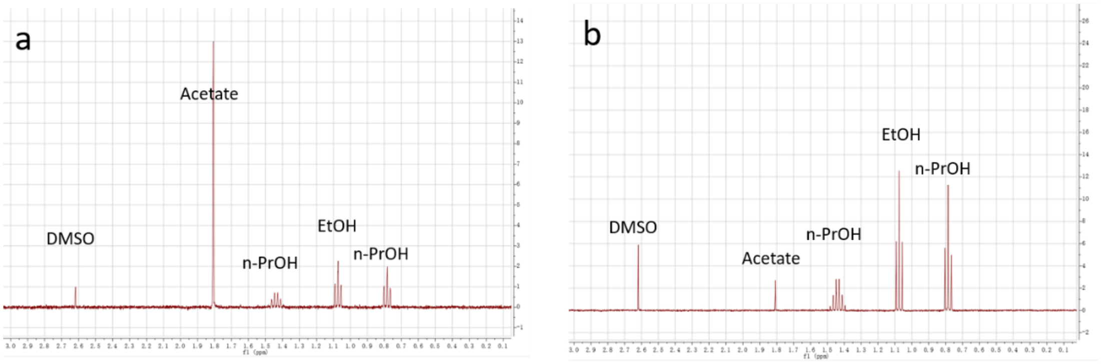

Supplementary Figure 40. NMR spectra of the liquid products. (**a**) ^{1}H NMR spectra of the liquid products from the Pb-Cu catalyst collected from the anode side during operation of CORR in a membrane electrode assemble (MEA) electrolyzer. (**b**) ^{1}H NMR spectra of the liquid products collected from the cathode side.

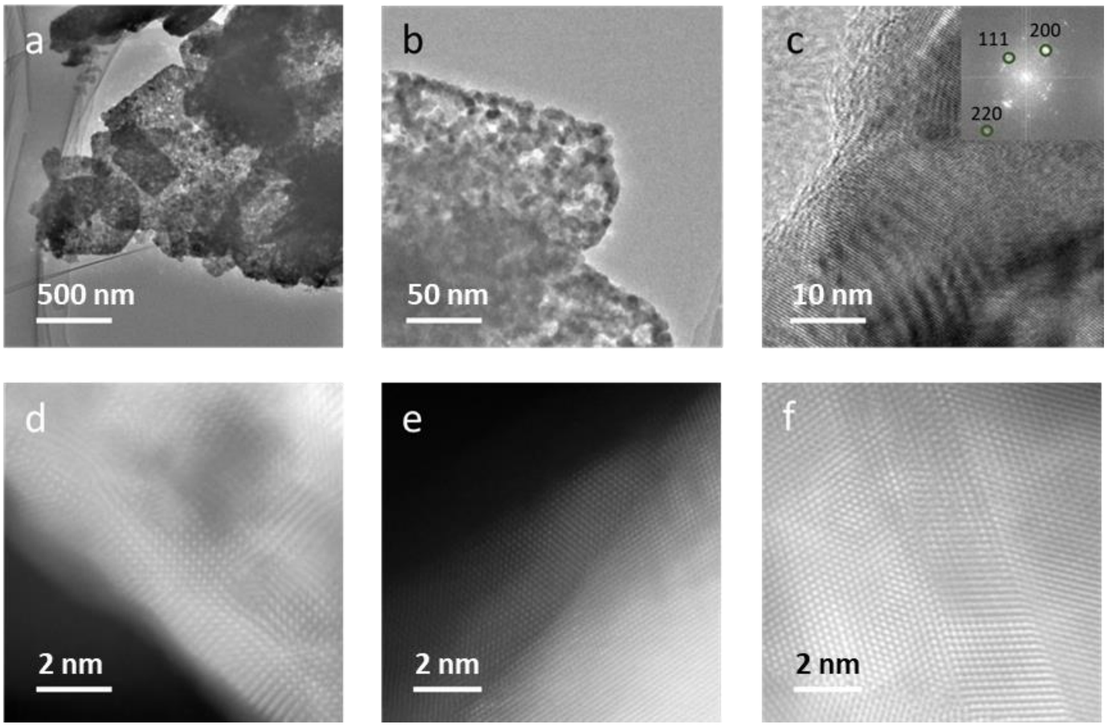

Supplementary Figure 41. TEM images of the Pb-Cu samples after the 100-h-stability test. (**a**, **b**) TEM images. Scale bar, 500 nm (**a**) and 50 nm (**b**). (**c**) HRTEM images of the Pb-Cu particles. Scale bar, 10 nm. Inset, the corresponding Fourier transfer image. (**d**-**f**) Atomic-resolution HAADF-STEM images of the Pb-Cu catalyst. Scale bar, 2 nm.

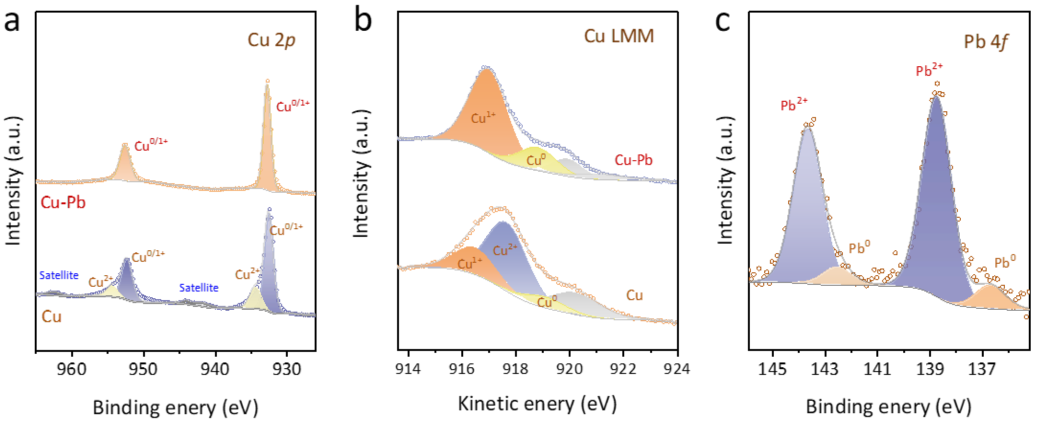

Supplementary Figure 42. X-ray photoelectron spectroscopy (XPS) spectra of the Pb-Cu electrodes after CORR. (**a**) High-resolution Cu 2*p*, (**b**) Cu LMM and (**c**) Pb 4*f* of the Pb-Cu catalysts. Cu 2*p* and Cu LMM Spectra of the Cu catalysts were listed for comparison.

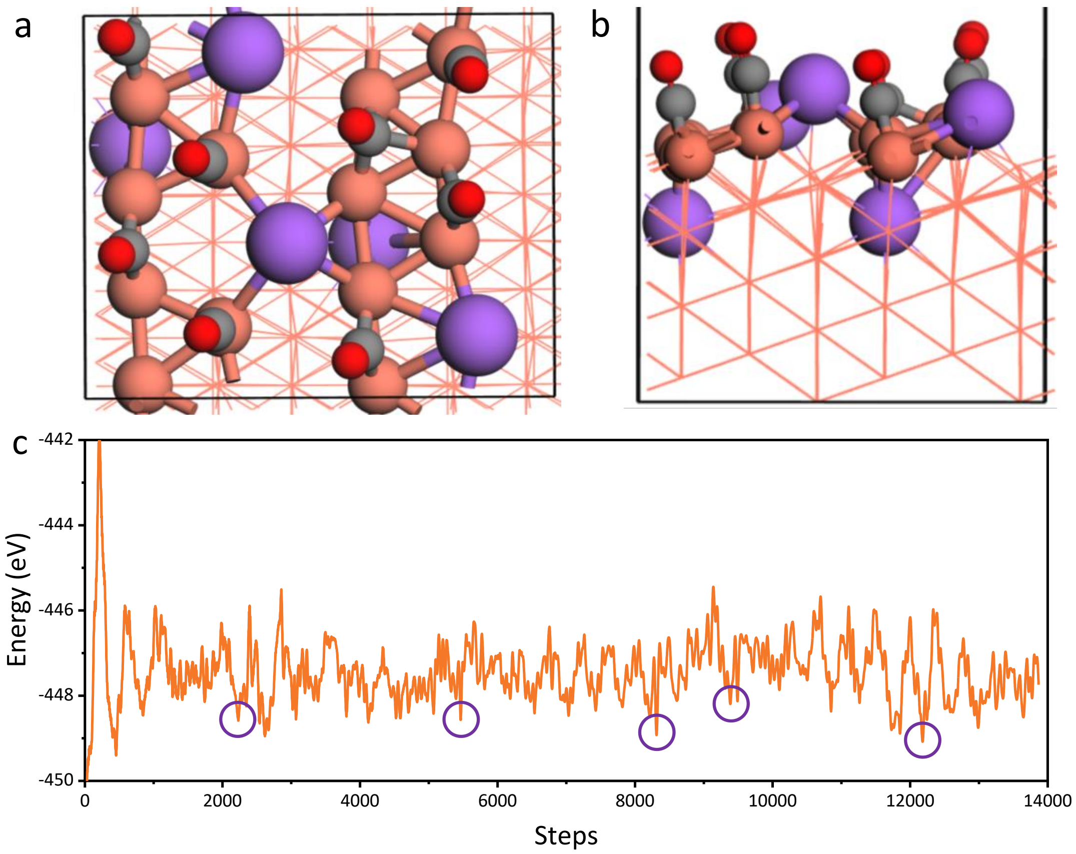

Supplementary Figure 43. AIMD simulations of the Pb-Cu surface. The (**a**) top view and (**b**) side view for the Pb doped Cu(211) surface, which is optimized by DFT calculation and used as the start point of MD simulation. (**c**) The MD simulation results, where the structures within purple circles were picked out. The grey, red, orange and purple balls (or lines) represents C, O, Cu and Pb, respectively.

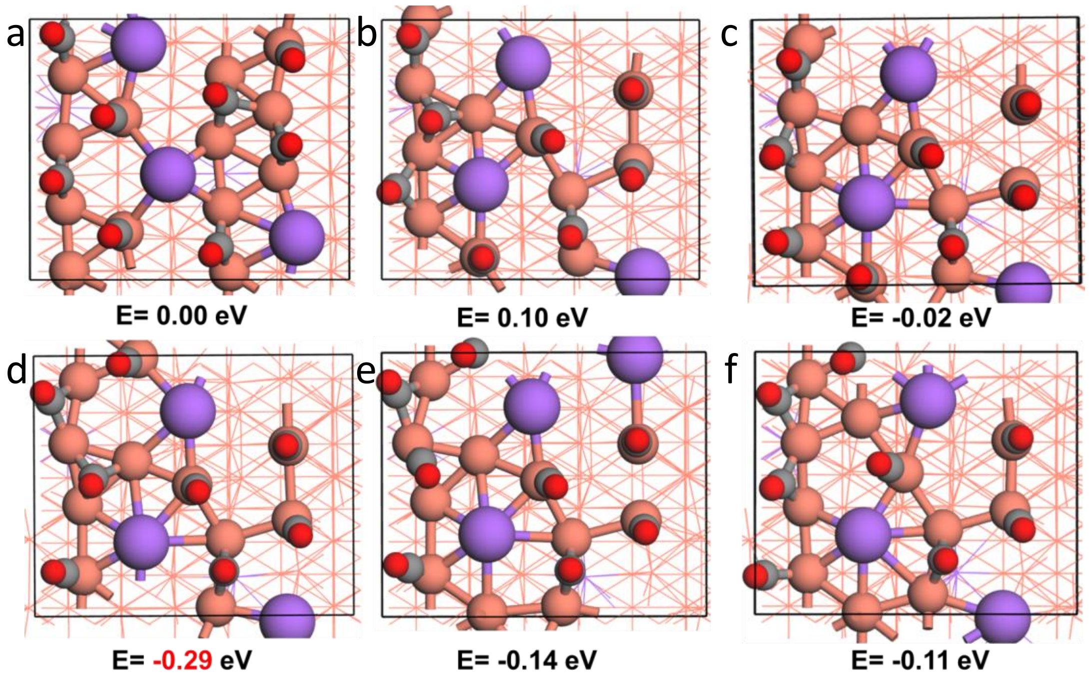

Supplementary Figure 44. The optimized structures picked out from the AIMD trajectory. The energy of the start point (Figure S36) is used as the zero point. The grey, red, orange and purple balls (or lines) represents the C, O, Cu and Pb, respectively.

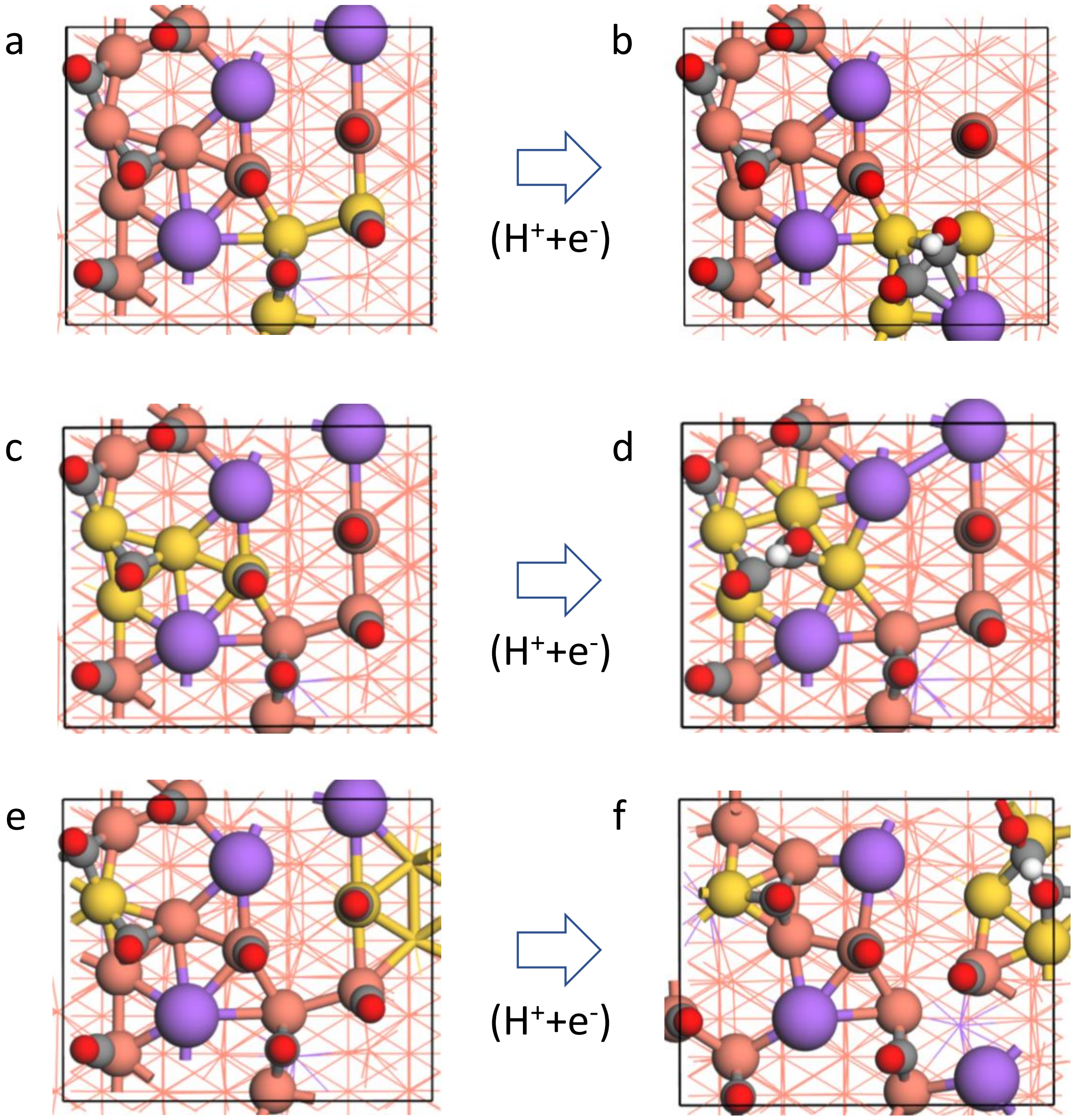

Supplementary Figure 45. The adaptive surface structure for *COCOH formation on the Pb-Cu surface. The structures before (**a**, **c**, **e**) and after (**b**, **d**, **f**) *COCOH formation on different sites are presented. The Cu atoms where *COCOH is formed are highlighted by the golden balls (surface atoms) or sticks (sub-surface atoms).

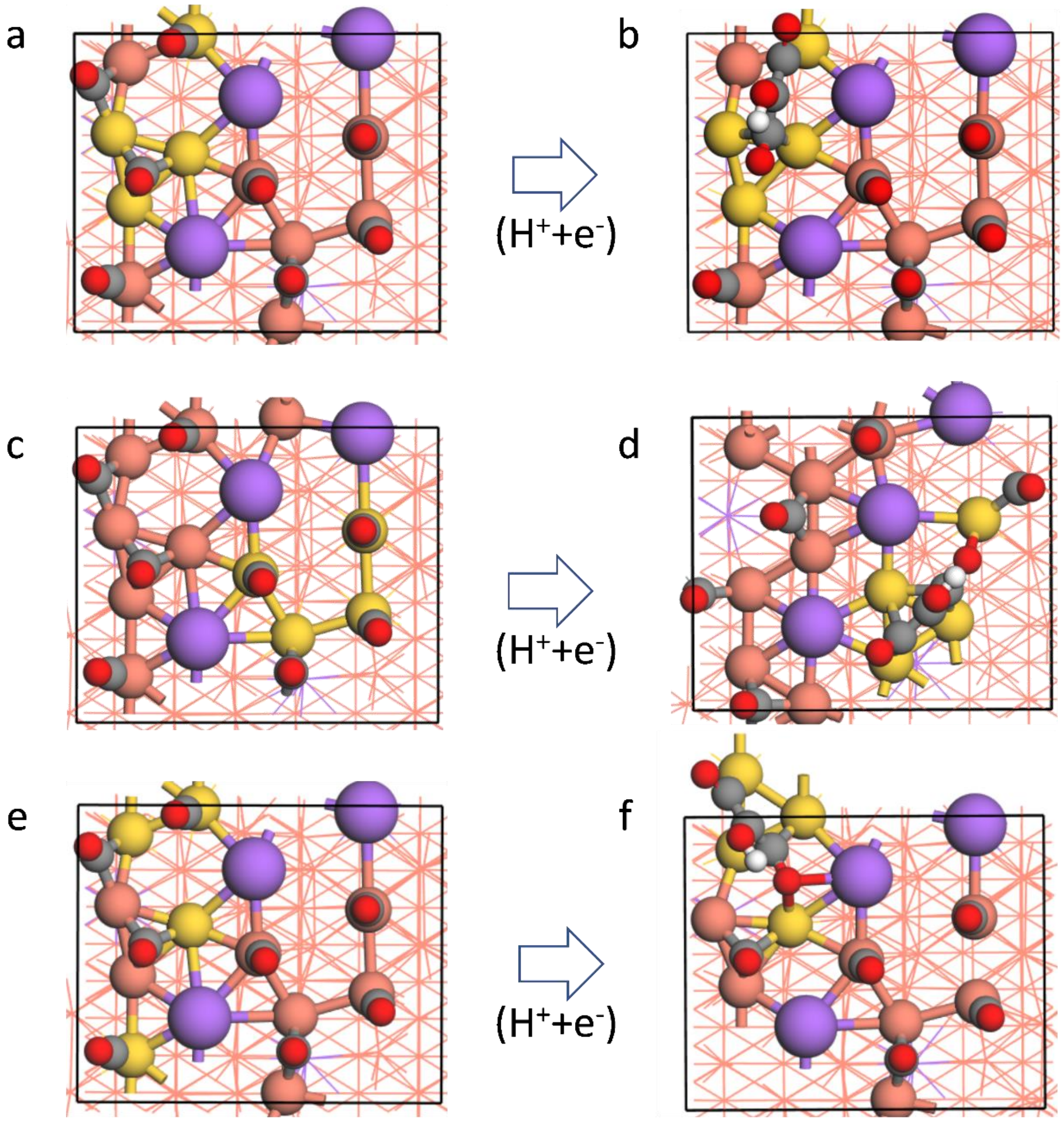

Supplementary Figure 46. The adaptive surface structure for *COCOHCO formation on the Pb-Cu surface. The structures before (**a**, **c**, **e**) and after (**b**, **d**, **f**) *COCOHCO formation on different sites are presented. The Cu atoms where *COCOHCO is formed are highlighted by the golden balls (surface atoms) or sticks (sub-surface atoms).

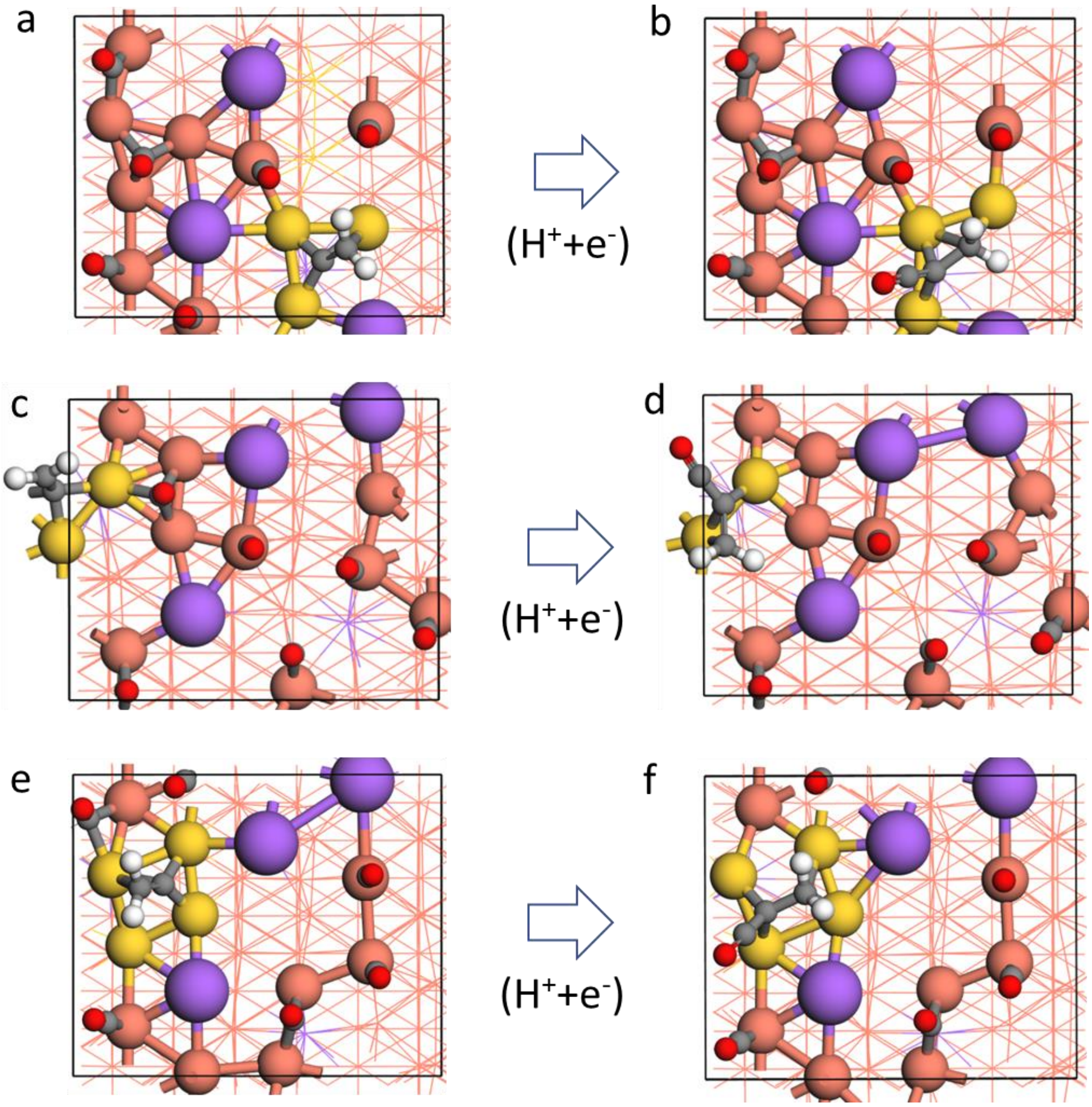

Supplementary Figure 47. The adaptive surface structure for *$COCCH_2$ formation on the Pb-Cu surface. The structures before (**a**, **c**, **e**) and after (**b**, **d**, **f**) * $COCCH_2$ formation on different sites are presented. The Cu atoms where *$COCCH_2$ is formed are highlighted by the golden balls (surface atoms) or sticks (sub-surface atoms).

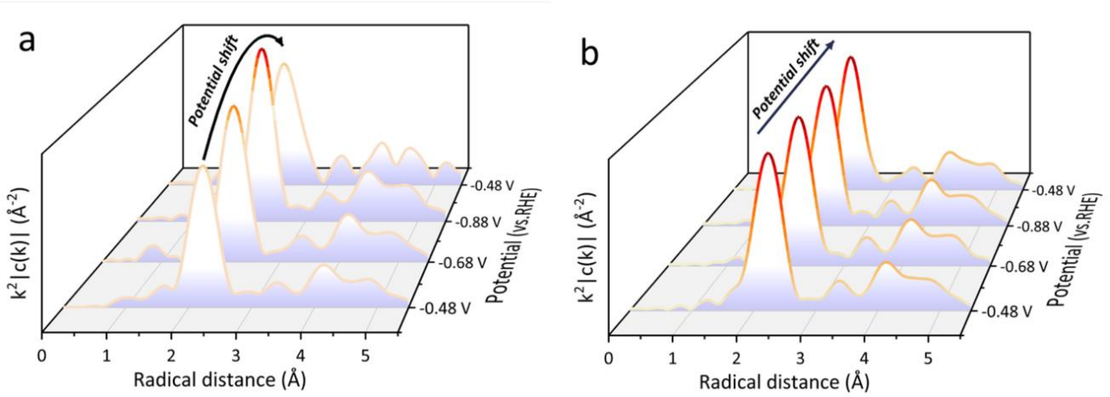

Supplementary Figure 48. Operando Cu K-edge EXAFS analyses at different potentials. Fourier-transformed $k^2\chi(k)$ of the Pb-Cu (**a**) and the Cu (**b**) catalysts with potential shift from -0.48 V to -0.88 V and backward to -0.48 V (*vs.* RHE).

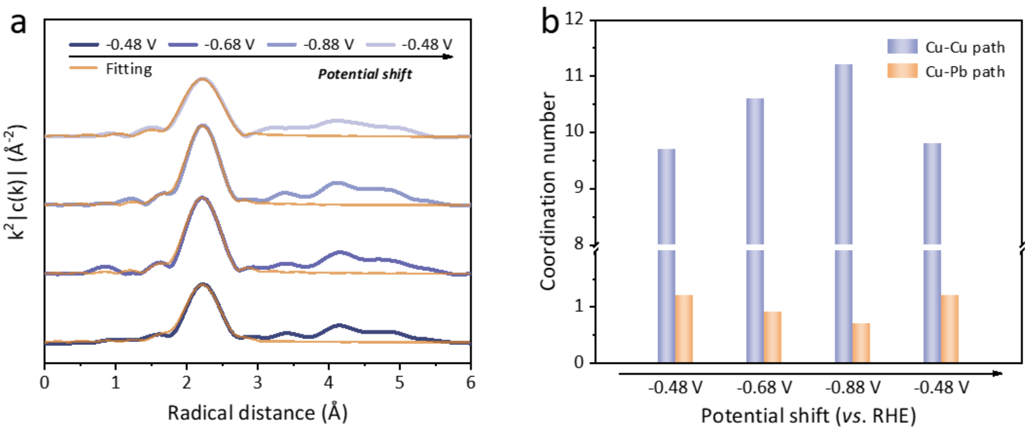

Supplementary Figure 49. Operando Cu K-edge EXAFS fitting analyses of the Pb-Cu catalysts. (**a**) Fourier-transformed $k^2\chi(k)$ of the Pb-Cu catalysts with the potential shift during CORR. (**b**) The coordination numbers of the Cu-Cu and the Cu-Pb paths under different potentials.

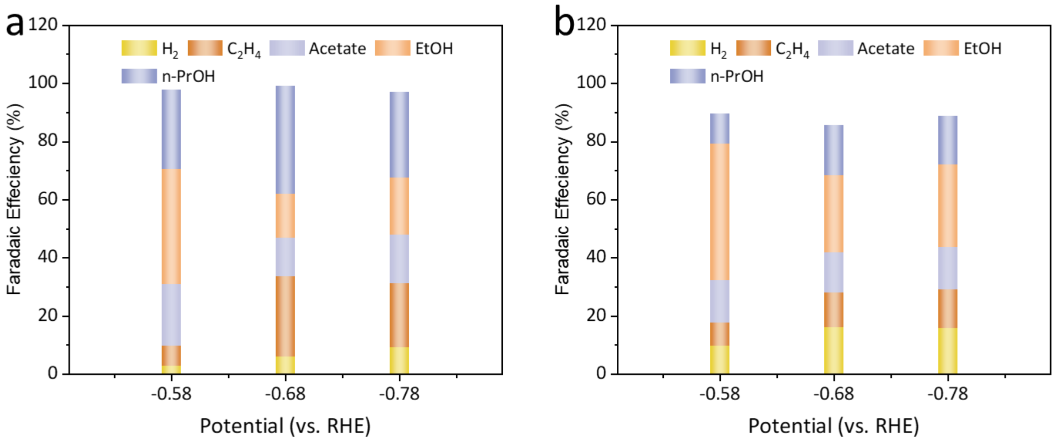

Supplementary Figure 50. CORR performances of catalysts in the ATR-SEIRAS cell. The FEs of the CORR products on (**a**) the Pb-Cu and (**b**) the Cu catalysts under different potentials.

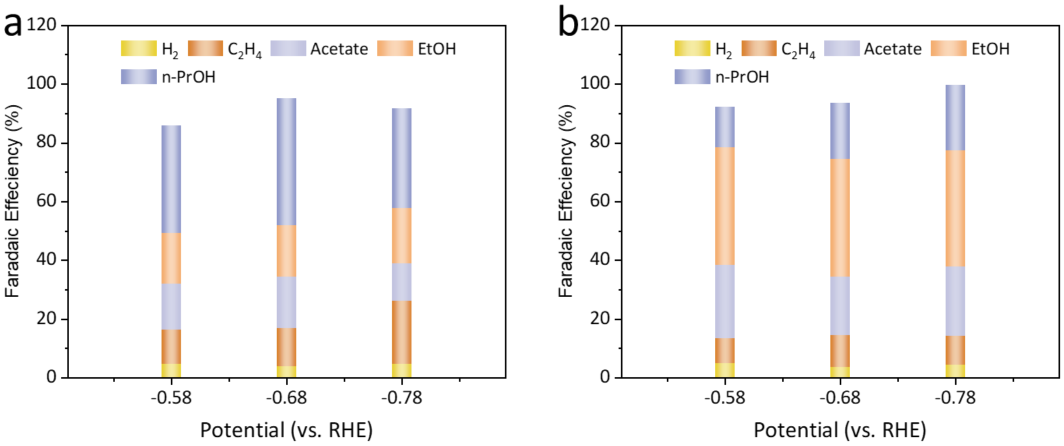

Supplementary Figure 51. CORR performances of catalysts in the Raman cell. The FEs of the CORR products on (**a**) the Pb-Cu and (**b**) the Cu catalysts under different potentials.

Supplementary Table 1. Elemental composition of Cu and Pb in different catalysts determined by inductively coupled plasma optical emission spectrometry (ICP-OES).

Sample	Element	Atom ratio (Cu : Pb)
Pb-Cu	Cu	1: 0.029
	Pb	
Pb-Cu-h	Cu	1: 0.036
	Pb	
Pb-Cu-l	Cu	1: 0.009
	Pb	

Supplementary Table 2. XPS results of different samples.

Sample	Element	Pb atoms ratio (%)
Pb-Cu	Cu	8.1
	Pb	
Pb-Cu-h	Cu	17.0
	Pb	
Pb-Cu-l	Cu	2.3
	Pb	

Supplementary Table 3. Fitting results of Cu K-edge EXAFS data of Cu, Pb-Cu, Pb-Cu-h and Pb-Cu-l catalysts[4].

Sample	EXAFS	bond	CN	CN_{total}	R (Å)	σ^2 (10^{-3} Å^2)
Cu	Cu K-edge	Cu-Cu	12	12	2.54	10.2
Pb-Cu		Cu-Cu	10.6	11.5	2.54	6.2
		Cu-Pb	0.9		2.73	
Pb-Cu-h		Cu-Cu	9.1	10.5	2,54	6.9
		Cu-Pb	1.4		2.81	
Pb-Cu-l		Cu-Cu	9.4	9.7	2.54	8.8
		Cu-Pb	0.3		2.76	

Supplementary Table 4. ECSA results of different samples[5].

Sample	Electric double-layer capacitance (mF)	Surface roughness factor
Com-Cu	9.77	336.9
Cu	12.94	446.2
Pb-Cu	8.85	305.2

Supplementary Table 5. A summary of Faradaic efficiencies for all products on the Pb-Cu catalyst in flow-cells.

Potential	Faradaic efficiency (%)					
(V vs. RHE)	H_2	C_2H_4	Acetate	Ethanol	n-Propanol	Total
-0.38	1.7	1.5±0.3	33.4±2.7	54.8±3.7	0	~91
-0.48	1.4	4.4±1.6	36.6±2.8	47.4±6.2	0	~90
-0.58	6.9	15.7±1.2	15.5±3.2	13.5±4.2	40.7±4.2	~92
-0.68	5.9	17.4±3.2	11.3±1.4	21.1±3.0	46.6±3.0	~102
-0.78	8.8	22.7±2.5	8.0±1.9	17.3±4.8	34.7±1.3	~92
-0.88	6.9	22.3±3.0	10.8±0.6	22.0±3.9	34.2±2.3	~96
-0.98	8.9	25.4±2.1	10.5±2.6	19.8±2.7	34.0±4.9	~99

Supplementary Table 6. A summary of Faradaic efficiencies for all products on the Cu catalyst in flow-cells.

Potential	Faradaic efficiency (%)					
(V vs. RHE)	H_2	C_2H_4	Acetate	Ethanol	n-Propanol	Total
-0.58	1.9	6.9±0.5	29.8±3.1	28.2±7.1	23.1±4.6	~90
-0.68	4.5	13.4±3.0	17.9±4.7	39.5±4.6	21.5±2.4	~97
-0.78	4.3	15.2±0.2	17.6±4.0	29.3±9.7	23.8±1.7	~90
-0.88	5.7	19.9±2.1	15.5±0.9	27.6±4.1	27.9±1.8	~97
-0.98	4.5	20.3±1.7	13.9±3.9	23.1±5.9	24.0±3.1	~86

Supplementary Table 7. Fitting results of Cu K-edge EXAFS data of the Pb-Cu catalysts under different potentials (V *vs.* RHE) [4].

Potential	EXAFS	bond	CN	CN_{total}	R (Å)	σ^2 (10^{-3} Å^2)
-0.48	Cu K-edge	Cu-Cu	9.7	10.9	2.54	7.3
		Cu-Pb	1.2		2.72	
-0.68		Cu-Cu	10.6	11.5	2.54	6.2
		Cu-Pb	0.9		2.73	
-0.88		Cu-Cu	11.2	11.9	2.54	6.2
		Cu-Pb	0.7		2.72	
-0.48		Cu-Cu	9.8	11.0	2.54	7.8
		Cu-Pb	1.2		2.72	

Supplementary Table 8. Fitting results of Cu K-edge EXAFS data of the Cu catalysts under different potentials (V *vs.* RHE)[4].

Potential	EXAFS	bond	CN	R (Å)	σ^2 (10^{-3} Å^2)
-0.48	Cu K-edge	Cu-Cu	12	2.54	9.8
-0.68		Cu-Cu	12	2.54	10.2
-0.88		Cu-Cu	12	2.54	9.9
-0.48		Cu-Cu	12	2.54	9.8

Supplementary Table 9. Summary of state-of-the-art CORR systems towards n-propanol.

Catalysts	FE (%)	*j* (mA cm^{-2})	Half-cell EE (%)*	Full-cell EE (%)	Stability	Cell type	References
**Pb-Cu**	**47**	**38**	**28**	N/A	**10 h**	flow-cell	**This work**
	**38**	**76**	N/A	**18**	**110 h**	MEA	**This work**
Ru-Ag-Cu	36	110	N/A	14	102 h	MEA	6
Ag-doped-Cu	33	4.5	21	N/A	N/A	flow-cell	7
Fragmented Cu	20	8.5	12	N/A	1.2 h	flow-cell	8
Nanocavity Cu	21	7.8	12	N/A	N/A	flow-cell	9
OD-Cu	14	20	N/A	N/A	N/A	flow-cell	10
BCF-Cu_2O	19	0.9	N/A	N/A	N/A	flow-cell	11
Cu adparticles	23	11	14	N/A	N/A	flow-cell	12

*The half-cell EEs are calculated by 70% iR compensation as the reference 7 for comparison.

References

1. Lv, X. *et al.* Electron-deficient Cu sites on Cu_3Ag_1 catalyst promoting CO_2 electroreduction to alcohols. *Adv. Energy Mater.* **10**, 2001987 (2020).
2. Mohanraju, K. & Cindrella, L. Impact of alloying and lattice strain on ORR activity of Pt and Pd based ternary alloys with Fe and Co for proton exchange membrane fuel cell applications. *RSC Adv.* **4**, 11939 (2014).
3. Li, J. *et al.* Constraining CO coverage on copper promotes high-efficiency ethylene electroproduction. *Nat. Catal.* **2**, 1124-1131 (2019).
4. Ravel, B. & Newville, M. ATHENA, ARTEMIS, HEPHAESTUS: data analysis for X-ray absorption spectroscopy using IFEFFIT. *J Synchrotron Radiat* **12**, 537-41 (2005).
5. Li, C.W., Ciston, J. & Kanan, M.W. Electroreduction of carbon monoxide to liquid fuel on oxide-derived nanocrystalline copper. *Nature* **508**, 504-7 (2014).
6. Wang, X. *et al.* Efficient electrosynthesis of n-propanol from carbon monoxide using a Ag–Ru–Cu catalyst. *Nat. Energy* **7**, 170-176 (2022).
7. Wang, X. *et al.* Efficient upgrading of CO to C_3 fuel using asymmetric C-C coupling active sites. *Nat. Commun.* **10**, 5186 (2019).
8. Pang, Y. *et al.* Efficient electrocatalytic conversion of carbon monoxide to propanol using fragmented copper. *Nat. Catal.* **2**, 251-258 (2019).
9. Zhuang, T.-T. *et al.* Copper nanocavities confine intermediates for efficient electrosynthesis of C_3 alcohol fuels from carbon monoxide. *Nat. Catal.* **1**, 946-951 (2018).
10. Jouny, M., Luc, W. & Jiao, F. High-rate electroreduction of carbon monoxide to multi-carbon products. *Nat. Catal.* **1**, 748-755 (2018).
11. Liu, J. *et al.* Directing the architecture of surface-clean Cu_2O for CO electroreduction. *J. Am. Chem. Soc.* **144**, 12410-12420 (2022).
12. Li, J. *et al.* Copper adparticle enabled selective electrosynthesis of n-propanol. *Nat. Commun.* **9**, 4614 (2018).
